# Traumatic brain injury epidemiology and rehabilitation in Ireland: a protocol paper

**DOI:** 10.12688/hrbopenres.13209.1

**Published:** 2021-06-15

**Authors:** Kate O'Donnell, Andrea Healy, Teresa Burke, Anthony Staines, Grainne McGettrick, Andrea Kwasky, Philip O'Halloran, Catherine Corrigan

**Affiliations:** 1 School of Nursing, Psychotherapy and Community Health, Dublin City University, Dublin 9, Ireland; 2 School of Psychology, Dublin City University, Dublin, Ireland; 3 Research and Policy Management, Acquired Brain Injury Ireland, Dun Laoghaire, Co Dublin, Ireland; 4 College of Health Professions and McAuley School of Nursing, University of Detroit Mercy, Detroit, Michigan, USA; 5 Royal College of Surgeons in Ireland, Dublin, Ireland; 6 Department of Neurosurgery, The Royal London Hospital, London, UK

**Keywords:** Traumatic Brain Injury, TBI, Head Injury, Brain Injury, Rehabilitation, Epidemiology, Health Services, Health Priorities

## Abstract

**Background:** Traumatic brain injury (TBI) is a leading cause of death and disability worldwide. In Ireland, a dearth of research on TBI means that we neither know the number of people affected by this injury, nor have the information required to improve neuro-rehabilitation services.

**Aims:** This is the protocol for a study that will examine pathways through rehabilitation for survivors of TBI in the Republic of Ireland. The experiences of family members providing care or support to a person with TBI will also be explored. Additionally, the study will estimate the incidence and prevalence of TBI in Ireland. Epidemiological data and information on how people with TBI access rehabilitation and health services will support advocacy efforts towards the redevelopment of neuro-rehabilitation services.

**Methods:** The research is a mixed method, observational cohort study design. Participants with moderate to severe TBI will be recruited through two brain injury service providers, two acute hospitals that provide neurosurgical services, and the National Rehabilitation Hospital. Questionnaires will be administered to participants with TBI on two separate occasions, six months apart, and to family members providing care or support to an individual with TBI, on one occasion. Data from the medical records of participants will be abstracted to capture key information about their brain injury. TBI survivor participants’ use of health care will be followed prospectively for six months.

**Expected outcomes:** The study will outline participants’ pathways through rehabilitation in Ireland, to understand how rehabilitation services are accessed, and the barriers to accessing these services. The incidence and prevalence of TBI in Ireland will be estimated. Experiences of family members providing care or support to an individual with TBI will be detailed. The outcomes of the study will support ongoing efforts to improve care for TBI survivors in Ireland and to redevelop neuro-rehabilitation services.

## Introduction

Traumatic brain injury (TBI) is the disruption to normal brain function caused by an exterior force or penetrating injury ^1^ and is a leading cause of death and disability worldwide ^2^. TBI can have long-term consequences, including a wide range of cognitive, sensory, behavioural, emotional and physical impairments for the individual, and social and socio economic ramifications for individuals, families, communities and societies ^1– 3^. A lack of formal surveillance or reporting systems for TBI has led to difficulties in establishing the magnitude of the problem; however, existing registries indicate that 7.7 million people in Europe and 5.3 million people in the United States (US) are living with disabilities related to TBI ^2, 3^. European incidence rates of TBI are estimated to be 262 per 100,000 population per year ^4, 5^, while United Kingdom (UK) incidence rates are estimated at 238 per 100,000 ^6^ and more than 2.5 million TBIs are recorded in the US each year ^1, 3^. In Ireland the incidence and prevalence of TBI is unknown and there is no national mechanism for “capturing the incidence, management and outcome of TBI presenting” to the health care system ^7^ (p.9). Integrated trauma systems are associated with decreases in trauma-related mortality and can facilitate clinical change ^8^. Decreases in mortality achieved through the implementation of an integrated trauma system and a trauma model of care delivery ^8^ will contribute to the demand for effective rehabilitation pathways in Ireland.

Evidence suggests that in adults with an acquired brain injury, intensive and early access to neuro-rehabilitation is cost-effective ^9, 10 ^ and promotes better outcomes ^11^. Timely access to acute care services can limit the impact of the primary head injury and its secondary complications, while access to ongoing rehabilitation can maximise functional recovery ^12^. The British Society of Rehabilitation Medicine (BSRM) guidelines for the management of rehabilitation following serious injury, propose that the rehabilitation pathway begins in the acute care phase of treatment ^13^. At this point, rehabilitation medicine consultants should identify the rehabilitation needs of the patient and direct them to appropriate rehabilitation services, expediting such referrals where necessary ^13^. The treatment setting is based on the complexity of the identified needs, with low complexity cases being treated on a local level ^13^. Turner-Stokes and colleagues’ review of multidisciplinary rehabilitation for adults with acquired brain injury (ABI) ^11^ suggested that the provision of rehabilitation services should be organised around need rather than diagnosis, and that the ongoing rehabilitation needs of an ABI survivor could be appropriately met in outpatient and community settings. Difficulties in accessing rehabilitation arise however, where there is a reliance on acute care practitioners to discharge patients to the appropriate clinical services ^14^ and a lack of organisation and systematic follow up in rehabilitative care ^15^. The patient’s geographical location, and health systems that require patients to self-advocate, also contribute to the experience of inequitable access to rehabilitation internationally ^12^.

In Ireland, issues of access to, and availability of, rehabilitation services reflect those experienced internationally. A lack of reliable data concerning patient groups who require neuro-rehabilitation undermines the planning of such services ^16, 17^. However, it is estimated that 40% of brain injury survivors will have a moderate to severe disability resulting in unmeasured personal, societal and economic consequences ^7^ and that 50,000 to 80,000 people with TBI need neuro-rehabilitation on an ongoing basis ^16^. Ireland has insufficient numbers of Physical Rehabilitation Medical Specialists (PRMS), with only nine out of the 27 PRMS recommended for Ireland’s population size ^18^. Reflecting the international situation ^14^, survivors of TBIs living in Ireland are often discharged from acute care facilities to inappropriate placements, such as nursing homes, or to their own homes, where rehabilitation services may not be available locally ^19^. Considerable gaps in rehabilitation-service provision, as well as excessively long delays in accessing services ^17, 20^ contribute to inappropriate placement ^19^. As is the case internationally, difficulty navigating the health system ^17, 20^ and poorly configured, inefficient, funding streams have been shown to relate to these gaps and delays ^19^. Geographically, there is disparity in service availability ^19^. Efforts to map this disparity have been complicated by the fact that referrals cannot be made to services that do not exist, creating difficulties in demonstrating demand ^19^. All of these issues influence the rehabilitation pathways of individuals with TBI in Ireland.

Lack of availability of rehabilitation services for people with TBI may result in significant burden to the family members of the TBI survivor. The transition from acute care to the community presents particular challenges for both individuals who have experienced a TBI and their families ^15, 21^. Responsibility to provide care frequently falls to the families of TBI survivors, who report feeling unprepared for the task ^15, 21^. Additionally, individuals with a brain injury may be discharged to home without an understanding (by themselves or their family members), of the long-term consequences of their injury ^20^. Delays in accessing rehabilitation may result in unnecessary disability ^20^ impacting rehabilitation potential and functional independence ^11^. Losses in functional independence, social networks, and occupational roles experienced by individuals with a TBI, can increase their reliance on family members ^20^, while changes in family roles can create tension and emotional difficulties ^21^. Financial pressures associated with loss of earnings and extra costs, such as housing adaptations and transport, add to the family burden ^21^. Findings of international studies indicate that family members often act as advocates and are vital to the long-term rehabilitation of individuals with TBI ^12, 15^. Furthermore, the availability of an advocate is a significant factor in successfully accessing rehabilitation services ^15^. The role and wellbeing of families and family caregivers are, therefore, important considerations in the rehabilitation pathways of individuals with TBI.

Ireland’s Neuro-rehabilitation Strategy ^22^ and subsequent implementation framework ^19^ seek to address deficits in rehabilitation provision through reconfiguring and integrating the systems that currently form the rehabilitation care pathway. An interdisciplinary approach to holistic rehabilitation, to be provided across the continuum of care, is proposed ^19^. Services will be accessible at four levels: primary care for lower level therapy needs; geographically based *Community Neuro-rehabilitation Teams*, providing specialist services to meet moderate therapy needs; regional neuro-rehabilitation services, accepting referrals from acute hospitals, specialist centres and community teams, to meet high level therapy needs; and national neuro-rehabilitation services, providing a high level of therapy for complex cases ^16, 19^. *Managed Clinical Rehabilitation Networks* will coordinate services to ensure timely and equitable access to rehabilitation ^19^. Despite this vision, progress with the implementation of the strategy is slow. Many of the concerns and challenges reflected in policy documents regarding the provision of neuro-rehabilitation published since 2001 continue to be key challenges today ^17^. These include a lack of epidemiological data and a lack of knowledge around the level of service need ^17, 19, 22^. Research in the UK has concluded that there is reasonably strong evidence to suggest long-term cost effectiveness of brain injury rehabilitation programmes ^23^. In Ireland, data is warranted to demonstrate the need for, and the effectiveness of, rehabilitation programmes for people with TBI ^17^.

It is expected that the findings of this research study will contribute to the literature on TBI in Ireland in a number of ways. Acknowledging the associated individual, societal and economic consequences ^1^, it will calculate the incidence and prevalence of moderate to severe TBI in Ireland, providing the epidemiological data to advance towards more effective systems of care and rehabilitation outlined in the Implementation Framework of the Health Service Executive (HSE) ^19^. The study will capture data on the mechanisms of injury in line with the classifications of the Phillips report ^7^. We will describe the pathways through rehabilitation, experienced by adults with moderate to severe TBI, in Ireland. Given previous research, we anticipate that the data will support findings of inequitable access to rehabilitation and variable outcomes for people with TBI in Ireland. The study will also record the current health service usage of individuals with TBI over a six-month period. We will capture individuals’ views on the benefits of rehabilitation received, as well as the unmet requirements on their rehabilitation journey. In addition to investigating the experiences of individuals with TBI in Ireland, and in recognition of the critical role played by families in influencing the rehabilitation pathways of individuals with TBI, this study will explore the experience of family members providing care or support to individuals with TBI. As some family members supporting an individual with TBI do not wish to be referred to as “caregivers” or “carers”, we will refer to both family caregivers and family members providing support to TBI survivors, simply as ‘family members’ throughout this paper.

The current study is undertaken in partnership between two leading Irish brain injury organisations, Acquired Brain Injury Ireland and Headway; two major trauma centres, Beaumont Hospital and Cork University Hospital; the National Rehabilitation Hospital; and Dublin City University (DCU). The study aims to document the incidence and prevalence of moderate to severe TBI in Ireland; describe patterns of disability associated with moderate to severe TBI; improve knowledge of rehabilitation pathways for TBI survivors in Ireland; assess the burden of TBI on family members, health services, and Irish society; and translate the research findings into a workable knowledge translation plan for TBI stakeholders. 

## Protocol

### Ethical approval

Ethical approval was granted by the DCU Research Ethics Committee (DCUREC/2018/123) and approval is sought by the ethics committees of all partner organisations through which participants are recruited: Acquired Brain Injury Ireland, Headway, Beaumont Hospital, Cork University Hospital, Letterkenny University Hospital and the National Rehabilitation Hospital.

### Primary and secondary aims

The primary aims of the study are:

- To describe the incidence, prevalence and patterns of disability associated with moderate to severe TBI survivors
- To improve the knowledge of rehabilitation pathways for TBI survivors
- To assess the burden on the carers, the health services, and Irish society
- To translate the research findings into a workable Knowledge Translation Plan for TBI stakeholders.

Secondary aims of the study are to develop and deliver on the Knowledge Translation Plan outlined below under ‘plans for dissemination’ and to disseminate the findings in conferences and publications.

### Study design

This is a quantitative, descriptive cohort study involving survivors of moderate to severe TBI and those who provide or have provided care for them. Cohort 1 will comprise TBI survivors that are at 3–12 months post injury and cohort 2 will comprise TBI survivors that are at least 12 months post injury. A cohort of people, who provide, or have provided care or support for TBI survivors will be recruited to form dyads. Participants with TBI will be surveyed on two separate occasions six months apart and followed-up monthly regarding their healthcare service use. Participants who care for TBI survivors will be surveyed on one occasion. Surveys will be completed, a) in person in a suitable location proposed by the participants, b) over the phone, or c) online. Data in relation to mechanism of injury, initial and long-term management, follow-up and referrals for further treatment or rehabilitation will be retrieved from medical records. The study will take place over a 30-month period beginning April 2019.

### Sampling plan

A purposive sampling method will be used to invite individuals with moderate to severe TBI to participate in the study. Following ethical approval, clinicians at partner sites (Acquired Brain Injury Ireland, Headway, The National Rehabilitation Hospital, Beaumont Hospital, Cork University Hospital and Letterkenny University Hospital) will identify potential participants who fit the inclusion criteria and invite them to participate in the research. The first cohort will be recruited through the acute hospitals, Beaumont Hospital, Cork University Hospital and Letterkenny University Hospital, and the second cohort will be recruited through the other partner sites. Individuals with TBI who are recruited to take part in the study will be asked to provide an invitation to a family member, or someone who provides them with care or support, to participate in the study. Two research assistants will collect informed consent from the participants for all three cohorts.

### Sample size calculation

Within the time and resources available for this project, we expect to recruit, and follow up, 100 TBI survivors in each cohort. This gives us sufficient power to estimate a true proportion of 0.5 within +/- 0.055, to estimate a mean to a precision of 0.07 standard deviations in each cohort, and to detect a difference between the means in the two cohorts of 0.35 standard deviations. Our judgement is that this is an adequate number of subjects to answer our key questions. We also aim to recruit one family member per TBI survivor recruited, who has provided, or provides, care or support to the person with TBI.

### Inclusion criteria

***Participants with TBI***

1. Individuals aged 18 years and above
2. Individuals who have sustained a moderate to severe TBI.

Injury severity will be determined as follows: ‘severe’ where a participant had a Glasgow Coma Scale (GCS) score of <9, loss of consciousness (LOC) for > 24 hours or post traumatic amnesia (PTA) lasting >1 week; ‘moderate’ where the participant had a GCS score between 9 and 12, LOC between 30 minutes and 24 hours or PTA that lasted between 24 hours and 1 week ^24^. If these measures of injury severity are not available, positive findings on computerised tomography (CT) or magnetic resonance imaging (MRI) will be used to determine injury severity.

3. Individuals who have capacity to give informed consent
4. Individuals who resident in Ireland
5. Individuals who sustained a TBI in the past 3 to 12 months (Cohort 1)
6. Individuals who sustained a TBI over 12 months ago (Cohort 2)

***Family member participants***

1. Individuals aged 18 years and above
2. Non-professional caregivers or family members who provide support to individuals with TBI.
3. Individuals who have capacity to give informed consent

As TBI is more prevalent in those younger than 25 years of age and older than 75 years of age, the study aims to capture data for adults with TBI without an upper age limit.

In order to be included in the study, participants will be required to have the capacity to give informed consent. In line with the principles of the Assisted Decision Making (Capacity) Act, 2015, participants will be assumed to have the capacity to give consent to participate in the study unless there is a reason to believe that they do not have the capacity to give consent ^25^. If a participant’s capacity to consent is in question, a clinician at the appropriate partner site will be asked to evaluate using the Functional Test for Capacity ^25^. This test of capacity is used to ascertain the participant’s (a) ability to understand; (b) at the time the decision has to be made; (c) the nature and consequences of the decision to be made; (d) in the context of available choices at the time ^25^.

### Exclusion criteria

***Individuals with TBI***

1. TBI survivors under the age of 18 years
2. TBI survivors with mild trauma (classified by GCS >12)
3. TBI survivors who do not reside in Ireland
4. TBI survivors who lack capacity to give informed consent to participate in this study at the time of recruitment.

***Family members***

1. Professional caregivers
2. Caregivers under the age of 18 years

### Data collection

Surveys will be administered to all participants, with options to complete them in-person or over the telephone in an interview format; independently online, or on paper. Individuals with TBI in each cohort will be surveyed at the point of inclusion into the study and again, approximately six months after the initial survey. Between the two surveys, participants will be asked to complete a monthly questionnaire about their use of health and rehabilitation services, allowing us to collect data relating to their ongoing rehabilitation. As participants in the TBI cohorts have moderate to severe brain injuries, we anticipate that many will opt to complete the survey with the support of a researcher in an interview format. Family members will be surveyed on one occasion. For family members supporting participants in the first cohort, this will take place close to the time of the second survey of the participant with TBI, to maximise the timeframe of experience providing care support. For family members of participants in the second cohort, surveys will be scheduled close to the first survey of the participant with TBI. In addition, the medical records of consenting participants with TBI will be abstracted to collect key data relating to their injury such as, the mechanism and severity, details of acute care and onward referrals for rehabilitation.

***Materials.*** Potential participants will be invited to participate in the study by the clinician, either in person, by telephone, or in written communication. An invitation pack comprising a letter of invitation, a patient information leaflet and a patient consent form will be provided to those invited to participate (see extended data ^26^). Potential participants will be encouraged to take time to decide whether they wish to be included in the study and to discuss this with someone they trust if they wish, before making a decision. Potential participants will also be encouraged to contact the research team with any questions they may have. 

A separate invitation pack for family members of the person with TBI will be included in the invitation pack sent to the potential participant with TBI. The individual with TBI will be requested to give this second pack to a family member who provides, or has provided care or support to them, and who may also wish to be involved in the study. This invitation pack will also comprise a letter of invitation, an information leaflet and a consent form.

The invitation packs to both the potential participant with TBI and the potential family member participant will include a postage-paid envelope for the return of the consent forms. Consent forms will be returned directly to the DCU research team. This process complies with General Data Protection Regulation (GDPR) 2016/679 ^27^ and will allow the research team to construct a database of participants. GDPR is a European Union (EU) data privacy and security law targeted at organisations collecting data relating to people in the EU. The research team will then take over responsibility regarding contact with the participants and surveys administration. Dyads of a participant with TBI and their family member will be matched from returned consent forms.

In addition, a plain-language project brochure calling for volunteers will be prepared for distribution in the partner organisations. A flyer will be prepared for distribution to professionals at relevant conferences and seminars, inviting their involvement in the project. Project information will be available on a website that was developed for the purpose of the study, and updates and news on the project will be shared regularly via Twitter. Participants may self-enrol through these avenues and will be included in the study if they meet the inclusion criteria.

### Outcome measures

Four questionnaires will be used for the research project. For the participants with TBI, an initial bespoke questionnaire will gather some personal and demographic information and information pertaining to the circumstances of the participant’s injury, employment and rehabilitation. Three standard instruments the EQ-5D-3L ^28^, WHOQOL BREF ^29^, European Brain Injury questionnaire (EBIQ) ^30^ will also be administered at this point. The follow up survey for participants with TBI will take place approximately six months after the first. A shorter version of the bespoke aspect of the first survey, designed to capture changes in living circumstances, employment and rehabilitation will be administered, and the three standard instruments will be repeated. Between the first and second surveys, participants with TBI will be asked to complete a short monthly survey to capture ongoing use of health and rehabilitation services (see extended data ^26^).

Family member participants will be asked to complete a bespoke questionnaire comprising questions that both compliment those administered to the participant with TBI, and elicit information concerning the family member participant. Questions will be asked in relation to the general circumstances, employment and rehabilitation of the person with TBI and the general circumstances, employment, care or support provided, and health of the family member. In addition, family member participants will be asked to complete three standard measures: Mayo-Portland Adaptability Inventory 4 ^31^, WHOQOL BREF ^32^, and the Burden Scale for Family Caregivers ^29^.

### Instruments

***EQ-5D-3L.*** The EQ-5D-3L (3 level version) is a widely used measure of health-related quality of life ^28^. It comprises a descriptive system and a visual analogue scale ^28^. The descriptive system outlines five dimensions (5D) of health: mobility, self-care, usual activities, pain/discomfort and anxiety/depression ^28^. Respondents are asked to indicate what they are experiencing with three levels (3L) of difficulty; for example, no problems, some problems, or extreme problems, on the day of administration ^28^. The vertical analogue scale displays a vertical measure from 0 to 100; where 0 is the ‘worst imaginable health state’ and 100 is the ‘best imaginable health state’ ^28^. The respondent is asked to indicate the point on this scale that best represents their health state on that day ^28^.

Health states are derived from the instrument by combining the values related to the level of problems experienced in each dimension, where the ‘no problems’ level is represented by 1, ‘some problems’ represented by 2 and the ‘extreme problems’ level is represented by 3 ^28^. For example, the health state of a person indicating no problems under mobility, some problems under self-care, some problems with usual activities, and extreme problems with both pain/discomfort and anxiety/depression, would be represented as ‘12233’ ^28^. The numbers assigned to the level of problem under a dimension have no arithmetic properties ^28^.

The EQ-5D instrument exists in two formats: EQ-5D-3L, outlined above, and the EQ-5D-5L which asks respondents to identify which of five levels of difficulty, under each of the same five dimensions of health as for the EQ-5D-3L, they are experiencing on the day of administration ^28^. Comparison of the EQ-5D-3L and the EQ-5D-5L suggests that the EQ-5D-3L is prone to ceiling effects and may not accurately discriminate problems experienced at the mild level, therefore demonstrating ‘full health’ where mild problems exist ^32^. One study examined the utility of adding a cognitive dimension to the EQ-5D-3L to increase the precision of the instrument in a TBI population but found that this added little explanatory power ^30^. Other criticisms of both the EQ-5D-3L and the EQ-5D-5L is that they are not sufficiently sensitive in capturing psychological or social dimensions ^30^. However, the EQ-5D-3L is widely used and is considered a credible basis for clinical decision making ^33^. An advantage of the EQ-5D-3L over other measures for health-related quality of life (HRQoL), is its brevity, and therefore low burden for completion ^30^. Permission to use this tool in this study was received from the Euroqol Research Foundation.

***WHOQOL-BREF.*** The World Health Organisation (WHO) defines quality of life as an ‘individual’s perception of their life in the context of the culture and value systems in which they live and in relation to their goals, expectations, standards and concerns’ ^29^ (p.1). Quality of life is ‘a broad ranging concept which is affected in a complex way by the person’s physical health, psychological state, level of independence, social relationships, personal beliefs and their relationship to salient features of their environment’ ^34^ (p.1). The WHOQOL-BREF is a 26-item abbreviation of the WHOQOL 100 that was developed using data from field trials in 15 countries ^29^. The original 100-item questionnaire produces scores relating to facets of quality of life and a score relating to overall quality of life and general health ^29^. The WHOQOL-BREF contains two items from overall quality of life and general health, and one item from each of the remaining 24 facets of the original 100 item measure, to yield domain scores in the areas of physical, psychological, social relationships and environment ^29^. The domain scores of the WHOQOL BREF have been shown to correlate at around 0.9 with the WHOQOL 100 domain scores ^29^. The WHOQOL instruments may be used in particular cultural settings but allow for cross-cultural comparisons as well ^29^. The WHOQOL-BREF can be used to provide insight into the effect of disease on subjective well-being ^29^. A study of the psychometric properties of the WHOQOL-BREF has demonstrated reliability and validity ^34^ and a systematic review supports its reliability, validity and responsiveness in a TBI sample ^35^. While there is a lack of consensus around health-related quality of life instruments suitable for use in people with TBI, studies support the applicability of the WHOQOL-BREF in this population ^36, 37^. Permission to use the WHOQOL-BREF in this research was obtained from World Health Organisation Press.

***European Brain Injury Questionnaire (EBIQ).*** The European brain injury questionnaire (EBIQ) was developed specifically for use with both brain injured patients and their relatives by Teasdale and colleagues ^38^. It comprises 63 questions regarding ‘problems or difficulties that people sometimes experience in their lives’. Respondents are requested to indicate if they have experienced these problems ‘not at all’, ‘a little’ or ‘a lot’, over the previous month ^38^. The questions can be grouped into nine domains or scales: somatic, cognitive, motivation, impulsivity, depression, isolation, physical, communication and core, all of which demonstrated satisfactory levels of reliability with Cronbach’s α value of near or above 0.5 ^38^. However, Martin *et al*. ^39^ clarify that only three of the nine scales - those relating to cognition, depression and impulsivity -demonstrate a high level of reliability. The scales demonstrating high reliability in the 1997 study by Teasdale and colleagues ^38^, were broadly similar to the results of a principal components analysis undertaken by Martin and colleagues ^39^ in a sample of people with TBI, which identified three factors: depression, cognitive difficulties and difficulties in social interaction. Sopena and colleagues ^40^ found that the EBIQ demonstrated robust test-retest reliability for persons with brain injury and relatives of persons with brain injury and Schonberger and colleagues ^41^ report r values of 0.47-0.66 for all scales with all p values <0.001. The EBIQ was designed for use with both people with brain injury and relatives of people with brain injury, in part on the basis that close relatives’ perspectives may balance lack of an awareness in the participant with TBI ^38, 40^. However, Martin and colleagues ^39^ argue that self-evaluation *is* a valid method of determining levels of difficulties experienced in daily life after severe TBI, and that this information cannot be obtained from interviewing a close relative. The current study will administer the EBIQ to participants with TBI only. The EBIQ is freely available and specific permission is not required to use the instrument for research purposes.

***Mayo-Portland Adaptability Inventory 4.*** The Mayo-Portland adaptability inventory-4 (MPAI-4) ^31^ has been developed from earlier versions, beginning with the Portland adaptability inventory developed by Lezak ^42^, to provide a meaningful documentation of the various cognitive, behavioural and social challenges experienced by those who have acquired a brain injury. The authors reported the use of Rasch analyses for improving and evaluating versions of the measure, describing how Rasch fit statistics guided selection of items and the development of rating and scoring procedures to maximise fit ^31^. The MPAI-4 includes 30 items covering limitations commonly experienced by those with an ABI. Items are rated on a five-point scale from 0-4, where 0 represents ‘normal’ function and 4 ‘severe limitations’. The instrument also comprises three subscales: the ability index, adjustment index and the participation index. The MPAI-4 demonstrates good levels of clinical utility and psychometric quality ^24^, with very good construct validity and internal consistency ^43^ in people with TBI. A recent study in an Irish sample with ABI reported very good internal consistency for the total scale score (0.91) as well as the three subscales: abilities (0.94), adjustment (0.82) and participation indices (0.85) ^37^. The MPAI-4 is freely available and specific permission is not required to use the instrument for research purposes.

***Burden Scale for Family Caregivers (BSFC).*** The Burden scale for family caregivers measure subjective burden in informal caregivers. It is available in 20 European languages, allowing for comparison between European populations ^44^. Subjective burden in those who provide care for the chronically ill has been found to significantly affect their emotional health, physical health and mortality as well as how the caregiver relates to the care receiver ^45^. The BSFC is a 28 item self-reporting instrument, that uses a four-point Likert scale ranging from ‘strongly agree’ to ‘strongly disagree’ ^45^. Split-half reliability test attained values of higher than 0.8 ^44^. The BSFC is freely available and specific permission is not required to use the instrument in research.

### Data management

Two research assistants will maintain a database on their encrypted, password protected computers. Hardcopy consent forms and survey responses will be stored separately and securely in locked cabinets in the offices of the researchers. Names and other contact details will be stored separately from completed questionnaires, be they on paper, or electronic format. Only the DCU research team will have access to the raw data. Unique identifiers will be used and no identifiable information will be published. The DCU Risk and Compliance Officer has reviewed a personal data security schedule (PDSS) that lists the categories of personal data being processed. Data is available in the Open Science Framework data repository. On completion of the study, the archived dataset will be anonymised and lodged with the Irish Social Science Data Archive (ISSDA).

### Data analysis and statistical plan

An analysis of the data generated throughout the study will be reported with input from the Knowledge Users (Acquired Brain Injury Ireland and Headway). Consultation with a Patient and Public Involvement (PPI) advisory panel and a Research Advisory Group, set up as part of the wider team involved in this study, will also inform reports.

Data points:

1. Hospitals’ and voluntary organisations’ medical record data of TBI participants
2. TBI participant surveys and six-month follow-up surveys
3. Family member survey data

Descriptive statistics using a range of univariate and multivariate statistical analyses will be employed to explore the data obtained through the partner sites and from participant surveys. Data will be analysed using statistical analysis software R ^46^ and SPSS version 27 ^47^.

We will examine the feasibility of sharing our anonymised data with the Irish Social Science Data Archive (ISSDA), the main Irish data repository. If a TBI registry is established in Ireland, anonymised data from this study will shared with registry developers.

### Reporting of results

Results of this research will be reported using the STROBE (STrengthening the Reporting of OBservational studies in Epidemiology) framework, a recommended checklist for reporting observational research ^48, 49^. We will report the number of individuals at each stage of study, for instance, the number potentially eligible; examined for eligibility; confirmed eligible; included in the study and completing follow-up. We will identify reasons for non-participation or attrition at each stage, and present these in a flow diagram. We will report on participant demographics, their clinical situation at admission and discharge from acute care, their pathway through rehabilitation and their use of health care over the duration of follow-up. We will report on participants’ financial situations, ways in which this has materially changed, their care situation, and the results of the standardised instruments.

### Ethics

Ethical approval will be obtained from Dublin City University, ABI Ireland, Headway, the National Rehabilitation Hospital, Beaumont Hospital and Cork University Hospital prior to the commencement of this research.

### Bias

There are several potential sources of bias in this study. The first is that the criteria for entry into the study are imperfect as there is no uniform data collection for people with TBI in Ireland. While every effort will be made to identify people with moderate to severe brain injury correctly, and to exclude those with mild brain injury and those with very severe and profound brain injury, this is believed to be imperfect. The group with the most severe disabilities, and who are most severely impacted will be excluded, as they would not be able to give informed consent. There are no independent central sources of information on rehabilitation services that can be used to check reported use. To mitigate this, service use will be ascertained from participants prospectively, which should minimise error.

### Dissemination and knowledge translation

A formal knowledge translation plan has been prepared with the Knowledge Users (ABI Ireland and Headway). The study has been designed in direct response to the needs of the TBI population identified by ABI Ireland and Headway Ireland, and findings will be applicable to these needs. The research protocol and conduct have been developed in a partnership with the researchers, Knowledge Users, the Public and Patient Involvement advisory panel and the Research Advisory Group.

The Knowledge Users are very experienced in managing political and policy advocacy campaigns and raising awareness of brain injury. The findings of this study will be directly applicable to these actions. In consultation with the PPI advisory panel, a plain language narrative synthesis of the research findings will be prepared and shared with key stakeholders. The research findings will also be shared with other organisations that may be able to use the data, for example, St. Doolagh’s Park Care and Rehabilitation Centre, Nua Healthcare, Redwood Extended Care Facility, The Irish Wheelchair Association, The Road Safety Authority and the Irish Medical Organisation. An open briefing will be held for TD’s, and senators in the Dáil.

The research team and the PPI panel will disseminate the final report. Advocacy efforts to influence health care pathways will be coordinated by ABI Ireland and Headway through the Neurological Alliance of Ireland (NAI). The NAI is instrumental in influencing health policy and practice on neuro-rehabilitation and has direct engagement with principal actors within the broader HSE clinical programme and the Department of Health. Knowledge Users, the researcher team and the Research Advisory Panel will collaborate on the basis of the data collected and PPI input, to propose solutions to the current waiting times for rehabilitation.

Knowledge Users and PPI advisory panel have made a number of specific suggestions, which are being, or will be implemented:

1. A social media strategy, with partner organisations to disseminate the findings to people with brain injuries, their families and the wider public
2. A launch seminar with all key stakeholders and other interested parties (for example, the Road Safety Authority, Irish Medical Organisation) to share findings
3. A Policy Briefing Paper to outline the policy issues that arise from the research conference dissemination
4. Presentations at Irish, European, and international conferences

Our findings will be submitted for publication to appropriate peer reviewed journals, such as Brain Injury, Neuro-epidemiology, The Journal of Head Trauma and Rehabilitation, and BMC Neurology.

There will be potential for further projects within the DCU/ Knowledge Users/ PPI partnership team, in particular around implementation of strategies, and evaluation of interventions.

### Study status

This study is well underway with data collection ongoing and the analysis of initial data currently in progress. It is expected that the research will be completed within the designated timeframe.

## Discussion

Advances in acute care have surpassed developments in rehabilitative care, resulting in increased demand for neuro-rehabilitation services ^19^, as more individuals who have experienced moderate to severe TBI are surviving. Increased demand, in turn, is contributing to longer waiting times for rehabilitation services, which are poorly configured to meet this demand ^19^. Previous research demonstrates that delayed rehabilitation can result in loss of function and unnecessary disability of TBI survivors ^19^, as well as pose significant challenges for their family members ^20^. The full scale of unmet need in Ireland is unknown to date ^7, 16^, and rehabilitation pathways for this population are essentially undocumented. The current study will address the current need for epidemiological data concerning TBI in Ireland and data on rehabilitation pathways for this population.

Ireland’s neuro-rehabilitation implementation plan outlines how rehabilitation services in Ireland might be reconfigured to achieve a flexible, responsive, accountable, rehabilitation service that can provide a standardised rehabilitation pathway ^19^. The service should be structured to deliver individualised rehabilitation locally, where possible, and in a timely and integrated manner, to meet the needs of service users ^19^. Through examining the rehabilitation pathways of individuals with moderate to severe TBI in Ireland, we expect that the current research findings will provide insight into the specific barriers to rehabilitation experienced by this population, and contribute valuable information to support the redevelopment of neuro-rehabilitation services. Additionally, increasing knowledge of the current rehabilitation pathways has the potential to positively impact outcomes for TBI survivors currently navigating the system. 

This study will be the first in Ireland to examine how individuals use healthcare services following a TBI; it will provide a comprehensive view on health services usage and the rehabilitation services required to support survivors of moderate to severe TBI. Information of this kind will support efforts to maximise health service availability for TBI survivors locally and nationally. The research will explore family members’ experiences of providing care and support to an individual with TBI in Ireland. Both international research and research within the Irish context demonstrate that there may be a considerable burden associated with providing care and support to TBI survivors ^15, 20, 21^. Understanding the considerable role of family members and informal carers in providing support to individuals with TBI to access services may be of particular importance to ensuring equity of access to rehabilitation ^15^. It is anticipated that a greater understanding of current rehabilitation pathways for TBI survivors in Ireland facilitated by this study may be of support to family members also.

A dearth of research in the area of TBI in Ireland means that we do not fully understand the difficulties faced by individuals with moderate to severe TBI in accessing rehabilitation services. Health policy documents dating back to 2001 have acknowledged the need to develop rehabilitation services and, more recently, a specific focus on neuro-rehabilitation services has found that services are inadequate and poorly configured to meet demand ^17, 19^. A key area of challenge identified is the lack of reliable data on the TBI population ^16, 17^. In this context, the current study is timely in its focus on the epidemiology of TBI in Ireland and on rehabilitation pathways for TBI survivors. It may contribute important information for the redevelopment of neuro-rehabilitation services. The findings of this study will be shared with our project partners to support the advocacy efforts of the brain injury organisations and to inform service providers and those attempting to access services, alike. As the first study of its kind in Ireland, it is anticipated that the findings will make a much-needed contribution to the Irish literature on TBI. 

## Data availability

### Underlying data

No data are associated with this article.

### Extended data

Open Science Framework: Traumatic Brain Injury - Pathways to rehabilitation. https://doi.org/10.17605/OSF.IO/2BAUF ^26^.

This project contains the following extended data:

- Carer-Family Member Questionnaire.pdf
- Participant materials HRB.pdf
- Person with TBI 1 ^st^ interview Questionnaire.pdf
- Person with TBI 2 ^nd^ Interview Questionnaire.pdf
- Person with TBI Health Care Usage.pdf

Data are available under the terms of the Creative Commons Attribution 4.0 International license (CC-BY 4.0).

## References

[1] Centers for Disease Control and Prevention: National Center for Injury Prevention and Control. 2019. Reference Source

[2] Rubiano AM Carney N Chesnut R : Global neurotrauma research challenges and opportunities. *Nature.* 2015;527(7578):S193–S197. 10.1038/nature16035 26580327

[3] Popescu C Anghelescu A Daia C : Actual data on epidemiological evolution and prevention endeavours regarding traumatic brain injury. *J Med Life.* 2015;8(3):272–277. 26351526 PMC4556905

[4] Brazinova A : Epidemiology of Traumatic Brain Injury in Europe: A Living Systematic Review. *J Neurotrauma.* 2015. 10.1089/neu.2015.4126 PMC8082737 26537996

[5] Peeters W van den Brande R Polinder S : Epidemiology of traumatic brain injury in Europe. *Acta Neurochir (Wien).* 2015;157(10):1683–1696. 10.1007/s00701-015-2512-7 26269030 PMC4569652

[6] Tenant A : Acquired Brain Injury: The Numbers Behind the Hidden Disability. 2018;9. Reference Source

[7] Phillips J : National Report on Traumatic Brain Injury in the Republic of Ireland 2008. 2008;67. Reference Source

[8] Deasy C Cronin M Cahill F : Implementing Major Trauma Audit in Ireland. *Injury.* 2016;47(1):166–172. 10.1016/j.injury.2015.07.016 26315666

[9] McGregor K Pentland B : Head injury rehabilitation in the U.K.: An economic perspective. *Soc Sci Med.* 1997;45(2):295–303. 10.1016/s0277-9536(96)00345-0 9225416

[10] Turner-Stokes L Dzingina M Shavelle R : Estimated Life-Time Savings in the Cost of Ongoing Care Following Specialist Rehabilitation for Severe Traumatic Brain Injury in the United Kingdom. *J Head Trauma Rehabil.* 2019;34(4):205–214. 10.1097/HTR.0000000000000473 30801440 PMC6687405

[11] Turner-Stokes L Pick A Nair A : Multi-disciplinary rehabilitation for acquired brain injury in adults of working age. *Cochrane Database Syst Rev.* 2015; (12):CD004170. 10.1002/14651858.CD004170.pub3 26694853 PMC8629646

[12] Copley A McAllister L Wilson L : We Finally Learnt to Demand: Consumers’ Access to Rehabilitation Following Traumatic Brain Injury. *Brain Impair.* 2013;14:436–449. 10.1017/BrImp.2013.32

[13] British Society of Rehabilitation Medicine: Rehabilitation for patients in the acute care pathway following severe disabling illness or injury: BSRM core standardsfor specialist rehabilitation. 2014;23. Reference Source

[14] Jourdan C Bayen E Bosserelle V : Referral to rehabilitation after severe traumatic brain injury: results from the PariS-TBI Study. *Neurorehabil Neural Repair.* 2013;27(1):35–44. 10.1177/1545968312440744 22460612

[15] Graff HJ Christensen U Poulsen I : Patient perspectives on navigating the field of traumatic brain injury rehabilitation: a qualitative thematic analysis. *Disabil Rehabil.* 2018;40(8):926–934. 10.1080/09638288.2017.1280542 28129694

[16] McGettrick G : National Policy and Strategy for the Provision of Neuro-Rehabilitation Services in Ireland 2011 – 2015.Policy Briefing Paper No. 2. 2015.

[17] Burke S McGettrick G Foley K : The 2019 neuro-rehabilitation implementation framework in Ireland: Challenges for implementation and the implications for people with brain injuries. *Health Policy.* 2020;124(3):225–230. 10.1016/j.healthpol.2019.12.018 31964508

[18] European Physical and Rehabilitation Medicine Bodies Alliance: White book on physical and rehabilitation Medicine (prM) in Europe. Chapter 5. The prM organizations in Europe: structure and activities. *Eur J Phys Rehabil Med.* 2018;54(2):198–213. 10.23736/S1973-9087.18.05149-3 29565106

[19] Health Services Executive: National Strategy and Policy for the Provision of Neuro-rehabilitation Services in Ireland. From Theory to Action. Implementation framework. 2019;96. Reference Source

[20] Muldoon OT Walsh RS Curtin M : Getting my life reset Living with an acquired brain injury: The Irish experience. 2017. Reference Source

[21] McDermott GL McDonnell AM : Acquired brain injury services in the Republic of Ireland: Experiences and perceptions of families and professionals. *Brain Inj.* 2014;28(1):81–91. 10.3109/02699052.2013.857790 24328803

[22] Department of Health: National Policy and Strategy for the Provision of Neuro-rehabilitation services. 2011;139. Reference Source

[23] Turner-Stokes L : The evidence for the cost-effectiveness of rehabilitation following acquired brain injury. *Clin Med (Lond).* 2004;4:10–12. 10.7861/clinmedicine.4-1-10 14998259 PMC4954264

[24] Kean J Malec JF Altman IM : Rasch Measurement Analysis of the Mayo-Portland Adaptability Inventory (MPAI-4) in a Community-Based Rehabilitation Sample. *J Neurotrauma.* 2011;28(5):745–753. 10.1089/neu.2010.1573 21332409 PMC3088961

[25] Assisted Decision-Making (Capacity) Act 2015.(Ireland) PT1. S.2, 3(2). Reference Source

[26] Staines A Corrigan C O'Donnell K : Traumatic Brain Injury - Pathways to rehabilitation.2021. 10.17605/OSF.IO/2BAUF

[27] Official Journal of the European Union: Regulations.2016. Reference Source

[28] The EuroQol Group: EuroQol--a new facility for the measurement of health-related quality of life. *Health Policy.* 1990;16(3):199–208. 10.1016/0168-8510(90)90421-9 10109801

[29] World Health Organization Division of Mental Health and Prevention of Substance Abuse: WHOQOL : measuring quality of life.12. Reference Source

[30] Geraerds AJLM Bonsel GJ Janssen MF : The added value of the EQ-5D with a cognition dimension in injury patients with and without traumatic brain injury. *Qual Life Res.* 2019;28(7):1931–1939. 10.1007/s11136-019-02144-6 30820809 PMC6571097

[31] Malec JF Kragness M Evans RW : Further Psychometric Evaluation and Revision of the Mayo-Portland Adaptability Inventory in a National Sample. *J Head Trauma Rehabil.* 2003;18(6):479–492. 10.1097/00001199-200311000-00002 14707878

[32] Thompson AJ Turner AJ : A Comparison of the EQ-5D-3L and EQ-5D-5L. *Pharmacoeconomics.* 2020;38(6):575–591. 10.1007/s40273-020-00893-8 32180162

[33] Berger-Estilita J Granja C Gonçalves H : A new global health outcome score after trauma (GHOST) for disability, cognitive impairment, and health-related quality of life: data from a prospective cross-sectional observational study. *Brain Inj.* 2019;33(7):922–931. 10.1080/02699052.2019.1581257 30810390

[34] Skevington SM Lotfy M O’Connell KA : The World Health Organization's WHOQOL-BREF quality of life assessment: psychometric properties and results of the international field trial. A report from the WHOQOL group. *Qual Life Res.* 2004;13(2):299–310. 10.1023/B:QURE.0000018486.91360.00 15085902

[35] Polinder S Haagsma JA van Klaveren D : Health-related quality of life after TBI: a systematic review of study design, instruments, measurement properties, and outcome. *Popul Health Metr.* 2015;13:4. 10.1186/s12963-015-0037-1 25722656 PMC4342191

[36] Chiu WT Huang SJ Hwang HF : Use of the WHOQOL-BREF for Evaluating Persons with Traumatic Brain Injury. *J Neurotrauma.* 2006;23(11):1609–1620. 10.1089/neu.2006.23.1609 17115908

[37] Fortune DG Walsh RS Waldron B : Changes in aspects of social functioning depend upon prior changes in neurodisability in people with acquired brain injury undergoing post-acute neurorehabilitation. *Front Psychol.* 2015;6:1368. 10.3389/fpsyg.2015.01368 26441744 PMC4561758

[38] Teasdale TW Christensen AL Willmes K : Subjective experience in brain-injured patients and their close relatives: a European Brain Injury Questionnaire study. *Brain Inj.* 1997;11(8):543–563. 10.1080/026990597123250 9251864

[39] Martin C Viguier D Deloche G : Subjective experience after traumatic brain injury. *Brain Inj.* 2001;15(11):947–959. 10.1080/02699050110065655 11689093

[40] Sopena S Dewar BK Nannery R : The European Brain Injury Questionnaire (EBIQ) as a reliable outcome measure for use with people with brain injury. *Brain Inj.* 2007;21(10):1063–1068. 10.1080/02699050701630342 17891569

[41] Schönberger M Humle F Teasdale TW : Subjective outcome of brain injury rehabilitation in relation to the therapeutic working alliance, client compliance and awareness. *Brain Inj.* 2006;20(12):1271–1282. 10.1080/02699050601049395 17132550

[42] Lezak MD : Relationships between personality disorders, social disturbances, and physical disability following traumatic brain injury. *J Head Trauma Rehabil.* 1987;2(1):57–69. 10.1097/00001199-198703000-00009

[43] Malec JF Kean J Altman IM : Mayo-Portland Adaptability Inventory: Comparing Psychometrics in Cerebrovascular Accident to Traumatic Brain Injury. *Arch Phys Med Rehabil.* 2012;93(12):2271–2275. 10.1016/j.apmr.2012.06.013 22743410

[44] Gräsel E : Somatic symptoms and caregiving strain among family caregivers of older patients with progressive nursing needs. *Arch Gerontol Geriatr.* 1995;21(3):253–266. 10.1016/0167-4943(95)00660-d 15374201

[45] Gräsel E Chiu T Oliver R : Development and Validation of the Burden Scale for Family Caregivers.2003;25. Reference Source

[46] R Core Team: R: A language and environment for statistical computing.R Foundation for Statistical Computing.2019.

[47] IBM Corp: IBM SPSS Statistics for Windows.Version 27.0. Armonk, NY: IBM Corp. Released2020.

[48] Lachat C Hawwash D Ocké MC : Strengthening the Reporting of Observational Studies in Epidemiology-Nutritional Epidemiology (STROBE-nut): An Extension of the STROBE Statement. *PLoS Med.* 2016;13(6):e1002036. 10.1371/journal.pmed.1002036 27270749 PMC4896435

[49] University of Bern: STROBE Statement. Strengthening the reporting of observational studies in epidemiology.2009. Reference Source

