# Traumatic brain injury epidemiology and rehabilitation in Ireland: a protocol paper

**DOI:** 10.12688/hrbopenres.13209.2

**Published:** 2022-09-22

**Authors:** Kate O'Donnell, Andrea Healy, Teresa Burke, Anthony Staines, Grainne McGettrick, Andrea Kwasky, Philip O'Halloran, Catherine Corrigan

**Affiliations:** 1 School of Nursing, Psychotherapy and Community Health, Dublin City University, Dublin 9, Ireland; 2 School of Psychology, Dublin City University, Dublin, Ireland; 3 Research and Policy Management, Acquired Brain Injury Ireland, Dun Laoghaire, Co Dublin, Ireland; 4 College of Health Professions and McAuley School of Nursing, University of Detroit Mercy, Detroit, Michigan, USA; 5 Royal College of Surgeons in Ireland, Dublin, Ireland; 6 Department of Neurosurgery, The Royal London Hospital, London, UK; 7 Centre for eIntegrated Care, Dublin City University, Dublin, 9, Ireland

**Keywords:** Traumatic Brain Injury, TBI, Head Injury, Brain Injury, Rehabilitation, Epidemiology, Health Services, Health Priorities

## Abstract

**Background:** Traumatic brain injury (TBI) is a leading cause of death and disability worldwide. In Ireland, a dearth of research means that we neither know the number of people affected by TBI, nor have the required data to improve neuro-rehabilitation services. This is a study protocol to examine the epidemiology and pathways through rehabilitation for a cohort of TBI survivors in the Republic of Ireland.

**Aims:**

1. To document the epidemiological data of TBIs in Ireland.

2. To explore the pathway of TBI survivors through rehabilitation/health services.

3. To document the experiences of those providing care for TBI survivors in Ireland

**Methods:** This is a quantitative cohort study. Existing routine datasets will be used to report epidemiological data. Participants with moderate or severe TBI will be recruited through two brain injury service providers, two acute hospitals that provide neurosurgical services, and the National Rehabilitation Hospital. Participants with TBI will be surveyed on two separate occasions, to explore their use of health and rehabilitation services. Those providing care or support to TBI survivors will be surveyed, on one occasion. Additionally, data from the medical records of TBI survivors will be extracted to capture key information about their TBI, such as mechanism of injury, severity, hospitalisation and follow-up. TBI survivors’ use of health care will be followed prospectively for six months.

**Expected outcomes:** The epidemiological data of TBI in Ireland will be documented. Data on survivors’ experiences of how rehabilitation services are accessed, and any barriers encountered with rehabilitation/health services will be reported. The experiences of those providing care or support for TBI survivors will be captured. It is expected that the outcomes of the study will support advocacy efforts toward the redevelopment of neuro-rehabilitation services in the Republic of Ireland.

## Introduction

Traumatic brain injury (TBI) is the disruption to normal brain function caused by an exterior force or penetrating injury ^1^ and along with spinal cord injury, is the largest cause of death and disability of trauma-related injuries worldwide ^2^. TBI can have long-term sequelae, including a wide range of cognitive, sensory, behavioural, emotional and physical impairments, and social and socio economic consequences for individuals, families, communities and societies ^1– 3^. A lack of formal surveillance or reporting systems for TBI has led to difficulties in establishing the magnitude of the problem in Ireland. In Europe, 7.7 million people and 5.3 million people in the United States (US) are living with disabilities related to TBI ^2, 3^. European incidence rates of TBI are estimated to be 262 per 100,000 population per year ^4, 5^, while the United Kingdom incidence rates are estimated at 238 per 100,000 ^6^ and more than 2.5 million TBIs are recorded in the US each year ^1, 3^. In Ireland the incidence and prevalence of TBI is unknown and there is no national mechanism for “capturing the incidence, management and outcome of TBI presenting” to the health care system ^7^ (p.9). Indeed, no unique ICD-10 code exists for TBI, making it difficult for epidemiologists to identify TBIs from routine data ^8^.

In Ireland, it is estimated that 40% of brain injury survivors will have a moderate or severe disability resulting in unmeasured personal, societal and economic consequences ^7^ and up to 150,000 people need neuro-rehabilitation on an ongoing basis ^9^. The international evidence ^10^ is reflected in Ireland where survivors of TBIs are often discharged from acute care facilities to inappropriate placements, such as nursing homes, or to their own homes, where rehabilitation services may not be available locally ^11^. There are considerable gaps in rehabilitation-service provision, as well as excessively long delays in accessing inappropriate services ^11^. A lack of reliable data concerning patient groups that require neuro-rehabilitation undermines the planning of such services ^9, 12^, for instance, there is disparity in service availability geographically ^11^. Efforts to map this disparity have been complicated by the fact that referrals cannot be made to services that do not exist, creating difficulties in demonstrating demand ^11^. Furthermore, Ireland has insufficient numbers of Physical Rehabilitation Medical Specialists with only 9 available of the recommended 27 PRMS for Ireland’s population size ^13^. All these challenges influence the rehabilitation pathways of individuals with TBI in Ireland.

Substantial economic consequences can be a major factor for brain injury survivors, their families and society. However, rehabilitation can improve the quality of life and reduce the hospital length of stay, therefore decreasing cost ^14^. In adults with severe brain injury, specialised rehabilitation is highly cost-efficient ^15^ and promotes better health outcomes ^16, 17^. Health ‘inequities’ can have noteworthy social and economic costs for individuals and societies as well. The World Health Organization defines health inequities as “systematic differences in the health status of different population groups” ^18(para 2)^. For example, in the context of TBI survivors, access to rehabilitation services is lower in rural areas, meaning that health inequities can occur because of inaccessibility ^19^. The patient’s geographical location, and health systems that require patients to self-advocate, also contributes to inequitable access to rehabilitation internationally ^19^.

Responsibility for the provision of care frequently falls to the families of TBI survivors, who report feeling unprepared for the task ^20, 21^. Individuals with a brain injury may be discharged home without an understanding by themselves or their family members, of the long-term consequences of their condition ^22^. This can result in a significant burden to family members. The transition from acute care to the community presents challenges ^20, 21^, for instance, delays in accessing rehabilitation may result in unnecessary disability ^22^ impacting rehabilitation potential and functional independence ^23^. Losses in functional independence, loss of social networks, and occupational roles, can increase the survivor’s reliance on family members ^22^, while changes in family roles can create tension and emotional difficulties ^21^. Financial pressures associated with loss of earnings and extra costs, such as housing adaptations and transport, add to the family burden ^21^. Findings from international studies indicate that family members often act as advocates and are vital to the long-term rehabilitation of individuals with TBI ^19, 20^. Furthermore, the availability of an advocate is a significant factor in successfully accessing rehabilitation services ^20^. The role and wellbeing of families and family caregivers are, therefore, important considerations in the rehabilitation pathways of individuals with TBI.

Timely access to acute care services can limit the impact to the patient, of the primary head injury and its secondary complications, while access to ongoing rehabilitation can maximise functional recovery ^19^. The British Society of Rehabilitation Medicine (BSRM) guidelines for the management of rehabilitation following serious injury, propose that the rehabilitation pathway begins in the acute care phase of treatment. At this point, rehabilitation medicine consultants should identify the rehabilitation needs of the TBI survivor and direct them to appropriate rehabilitation services, expediting such referrals where necessary. The treatment setting is based on the complexity of the identified needs, with low complexity cases being treated on a local level ^24^. Difficulties in accessing rehabilitation arise however, where there is a reliance on acute care practitioners to discharge patients to the appropriate clinical services ^10^ and a lack of organisation and systematic follow-up in rehabilitative ensues ^20^.

Rehabilitation typically focuses on the restoration of motor and functional recovery to the same level of function as prior to the injury ^25^. Rehabilitation can be non-specialised (motor and functional activities), or specialised, for instance a cognitive rehabilitation program ^17^. Rehabilitation pathways provide a streamlined approach to the appropriate service, ideally involving an interdisciplinary team within a multi-service system. Integrated trauma systems (across services and multidisciplinary teams) are associated with decreases in trauma-related mortality, can facilitate clinical change ^16^ and contribute to the demand for effective rehabilitation pathways. Turner-Stokes and colleagues’ review of multidisciplinary rehabilitation for adults with acquired brain injury (ABI) ^23^ suggested that the provision of rehabilitation services should be organised around need rather than diagnosis, and that the ongoing rehabilitation needs of an ABI survivor could be appropriately met in outpatient and community settings.

Ireland’s Neuro-rehabilitation Strategy ^26^ and subsequent implementation framework ^11^ address deficits in rehabilitation provision through reconfiguring and integrating the systems that currently form the rehabilitation care pathway. An interdisciplinary approach to holistic rehabilitation, to be provided across the continuum of care, is proposed by the Health Service Executive (HSE) ^11^. The strategy includes accessibility at four levels: primary care for lower level therapy needs; geographically based *Community Neuro-rehabilitation Teams*, providing specialist services to meet moderate therapy needs; regional neuro-rehabilitation services, accepting referrals from acute hospitals, specialist centres and community teams, to meet high level therapy needs, and national neuro-rehabilitation services, providing a high level of therapy for complex cases ^9, 11^. *Managed Clinical Rehabilitation Networks* will coordinate services to ensure timely and equitable access to rehabilitation ^11^. Despite this vision, progress with the implementation of the strategy is slow and indeed, many concerns and challenges reflected in neuro-rehabilitation policy documents published since 2001 continue to be key challenges today ^12^. These include a lack of epidemiological data and a lack of knowledge around the level of service need ^11, 12, 26^. In Ireland, data is warranted to demonstrate the need for, and the effectiveness of, rehabilitation programmes for people with TBI ^12^.

It is expected that the findings from this research study will contribute to the literature on TBI in Ireland in a number of ways. The research team aims to document the epidemiological data of moderate or severe TBI, describe patterns of associated disability, and document the rehabilitation experiences of TBI survivors in Ireland. We will also assess the burden of TBI on family members, health services, and the Irish society, and translate the research findings into a workable knowledge translation plan for TBI stakeholders.

Providing epidemiological data of TBIs in Ireland and reporting on the individual, family, societal and economic consequences ^1^ of moderate or severe TBI, will help to advocate for effective systems of care and rehabilitation outlined in the Implementation Framework of the Health Service Executive (HSE are the national public health service in Ireland) ^11^. The team will capture data on the mechanisms of injury in line with the classifications of the Phillips report ^7^ and outline the pathways through rehabilitation, experienced by adults with moderate or severe TBI. In line with previous research, we anticipate that the data will support findings of inequitable access to rehabilitation and variable outcomes for TBI survivors. We will also record the health service usage of individuals with TBI over a six-month period and capture individuals’ views on the benefits of rehabilitation received, as well as the unmet requirements on their rehabilitation journey. In addition to investigating the experiences of individuals with TBI, and in recognition of the critical role played by families in influencing the rehabilitation pathways of TBI survivors, we will explore the experience of those providing care or support to individuals with TBI. We will refer to family caregivers and others providing support or care, simply as ‘carers’.

This study is in process, in partnership with two leading Irish brain injury organisations, Acquired Brain Injury Ireland and Headway; two major trauma centres, Beaumont Hospital and Cork University Hospital; the National Rehabilitation Hospital and Dublin City University (DCU).

## Protocol

### Ethical approval

Ethical approval was obtained from the Research Ethics Committee in DCU (DCUREC/2018/123) and the ethics committees of all partner organisations: Acquired Brain Injury Ireland, Headway, Beaumont Hospital, Cork University Hospital, and the National Rehabilitation Hospital.

### Primary and secondary aims

The primary aims of the study are:

- To describe the incidence, prevalence and patterns of disability associated with moderate or severe TBI survivors
- To improve the knowledge of rehabilitation pathways of TBI survivors
- To assess the burden on the carers, the health services, and the Irish society
- To translate the research findings into a workable Knowledge Translation Plan for TBI stakeholders.

Secondary aims of the study are to develop and deliver on the Knowledge Translation Plan and to disseminate the findings in conferences and publications globally.

### Study design

This is a quantitative, cohort study involving survivors of moderate or severe TBI and those who provide, or have provided care for them. Cohort 1 will comprise TBI survivors that are at 3–12 months post injury; cohort 2 will comprise TBI survivors that are 12 months or longer post injury. A cohort of carers will be recruited to form dyads. Participants with TBI will be surveyed on two separate occasions six months apart and followed-up monthly regarding their health care service use. Some qualitative data may be derived from the monthly account of current health service usage which we will report if deemed useful in response to the aims of the study. Carers will be surveyed on one occasion. Surveys can be completed, a) in person in a suitable location proposed by the participants, b) over the phone, or c) online. We anticipate that TBI survivors will need assistance with the questionnaire which will be provided by the research team. Data in relation to mechanism of injury, initial and long-term management, follow-up and referrals for further treatment or rehabilitation will be retrieved from the medical records. The study, initially planned for a 30-month period, has already begun. Of note however, are the challenges in data collection brought on by Covid-19. It was difficult to identify participants as the research team were unable to access consent forms from the postal service which was the main method of participant identification. Lockdown in March 2020 coincided with hospital recruitment of participants. A project website was developed in an effort to promote recruitment with minimal benefit, nevertheless, the study continues with a sufficient number of respondents.

In addition to the study participant data, existing routine datasets will be accessed:

a) Major Trauma Audit (MTA) ^16^ records the rehabilitation recommendations for all trauma patients following the acute phase of their injury. We will collect data on the prevalence of TBI causalities, the number of TBI survivors who require rehabilitation and the level of rehabilitation recommended. A limitation is that MTA is only in existence since 2014 and their data capture is not 100% (currently estimated at 55–60%).

b) Hospital In-Patient Enquiry (HIPE) data captures demographics, clinical data and deaths from all the acute public hospitals in Ireland. HIPE reports over 1.5 million records annually. through Health Intelligence, HSE. We will collect data on discharges and morbidity and mortality TBI data. HIPE data is reported in aggregate format, meaning that linking it to the patients in the research study is not possible; however, we can use it in addition to the cohorts we intend to study to report our findings in context.

c) The National Physical and Sensory Disability Database (NPSDD) is a service-planning tool that provides a profile of people with physical or sensory disability (approx. 23,000 records created annually) and data on current service use regionally and nationally. Expected future service needs for people with physical or sensory disability are also recorded. NPSDD focuses on physical/sensory disability in general, meaning that data pertaining to TBI specifically is not retrievable. Registration on the database is voluntary, meaning that the numbers are not definitive; nevertheless, we will access the data for information on current service use in Ireland.

d) Central Statistics Office will be used to extract national mortality data. Specific TBI case fatality data are not available; age specific mortality data with relevant external causes of morbidity and mortality codes, along with data from this study will assist in estimating numbers. 

### Sampling plan

A purposive sampling method will be used to invite individuals with moderate or severe TBI to participate in the study. Clinicians at partner sites (Acquired Brain Injury Ireland, Headway, Beaumont Hospital, Cork University Hospital, National Rehabilitation Hospital,) will identify potential participants who fit the criteria and invite them to participate in the study. The first cohort will be recruited through the acute hospitals, Beaumont Hospital and Cork University Hospital; the second cohort will be recruited through the other partner sites. Individuals with TBI who are recruited to take part in the study will be asked to provide an invitation pack to a carer who provides care or support for them, to participate in the study.

### Sample size calculation

Within the time and resources available for this project, we expect to recruit, and follow up, 100 TBI survivors in each cohort. This gives us sufficient power to estimate a true proportion of 0.5 within +/- 0.055, to estimate a mean to a precision of 0.07 standard deviations in each cohort, and to detect a difference between the means in the two cohorts of 0.35 standard deviations. Our judgement is that this is an adequate number of subjects to answer our key questions. We also aim to recruit one carer per TBI survivor recruited, who has provided, or provides, care or support to the person with TBI.

### Inclusion criteria

***Participants with TBI***

- Individuals aged 18 years and above
- Individuals who have sustained a moderate or severe TBI
- Individuals who have capacity to give informed consent
- Individuals who resident in Ireland

Injury severity will be determined as follows: ‘severe’ where a participant had a Glasgow Coma Scale (GCS) score of <9, loss of consciousness (LOC) for > 24 hours or post traumatic amnesia (PTA) lasting >1 week; ‘moderate’ where the participant had a GCS score between 9 and 12, LOC between 30 minutes and 24 hours or PTA that lasted between 24 hours and 1 week ^27^. If these measures of injury severity are not available, positive findings on computerised tomography (CT) or magnetic resonance imaging (MRI) will be used to determine injury severity.

***Carer participants***

- Individuals aged 18 years and above
- Non-professional caregivers who provide support to individuals with TBI

In order to be included in the study, participants will be required to have the capacity to give informed consent. In line with the principles of the Assisted Decision Making (Capacity) Act, 2015, participants will be assumed to have the capacity to give consent unless there is a reason to believe that they do not have the capacity to give consent ^28^. If a participant’s capacity to consent is in question, a clinician at the appropriate partner site will be asked to evaluate using the Functional Test for Capacity. This test of capacity is used to ascertain the participant’s (a) ability to understand; (b) at the time the decision has to be made; (c) the nature and consequences of the decision to be made; (d) in the context of available choices at the time ^28^.

### Exclusion criteria

***Individuals with TBI***

- TBI survivors with mild trauma (classified by GCS >12, LOC <30 min, and PTA lasting <24 hours) ^27^
- TBI survivors who lack capacity to give informed consent at the time of recruitment.

***Carers***

- Professional caregivers

### Data collection

Surveys will be administered to all participants, with options for completion in-person, by phone, online, or on paper. Individuals with TBI in each cohort will be surveyed at the point of inclusion into the study and approximately six months after the initial survey. As participants have moderate or severe brain injuries, we anticipate that many will opt to complete the survey with the support of a researcher in an interview format. Between the two surveys, participants will be asked to complete a monthly questionnaire about their use of health and rehabilitation services. In addition, the medical records of consenting TBI survivor participants will be accessed to collect key data relating to their injury such as, mechanism and severity, details of acute care and referrals for rehabilitation. Carers will be surveyed on one occasion.

***Materials.*** Potential participants will be invited to participate in the study by the clinician, either in person, by telephone, or by written communication. An invitation pack comprising a letter of invitation, an information sheet and a consent form will be provided to those invited to participate (see extended data ^29^). Potential participants will be encouraged to take time to decide if they wish to be included in the study, to discuss the research with someone they trust, and to contact the research team with questions if they wish, before making a decision.

A separate invitation pack for carers will be included in the TBI survivors’ invitation pack. The TBI survivor will be asked to give this second pack to a person who provides, or has provided care or support for them. 

The invitation packs to both the potential participant with TBI and their carer include a postage-paid envelope for the return of the consent forms directly to the DCU research team. This process complies with General Data Protection Regulation (GDPR) 2016/679 ^30^ and will allow the research team to construct a database of participants. GDPR is a European Union (EU) data privacy and security law targeted at organisations collecting data relating to people in the EU. Dyads of participants with TBI and their carers will be matched from returned consent forms.

In addition, a plain-language brochure calling for volunteers will be distributed to the partner organisations. Flyers will also be distributed to professionals at relevant conferences and seminars, inviting their involvement in the project. Project information will be available on a research project website, and updates and news on the project will be shared regularly via Twitter. Participants may self-enrol through these avenues and will be included in the study if they meet the criteria.

### Outcome measures

Four questionnaires will be used for this project. TBI survivors will be asked to complete a bespoke questionnaire to collect demographic data, and data pertaining to the circumstances of their injury, employment and rehabilitation. Three standard instruments the EQ-5D-3L, WHOQOL BREF, and the European Brain Injury questionnaire (EBIQ) will also be administered. The follow-up survey for participants with TBI will be administered approximately 6 months after the first survey. A shorter version of the bespoke questionnaire, designed to capture changes in living circumstances, employment and rehabilitation, and the three standard instruments will be repeated. Between the first and second surveys, TBI survivors will be asked to complete a short monthly survey to capture their ongoing use of health and rehabilitation services (see extended data ^29^).

Carer participants will be asked to complete a bespoke questionnaire comprising questions that compliment those administered to the TBI survivor. The questions will include items relating to the TBI survivor, for example, general circumstances, employment and rehabilitation and items concerning the carer such as, general circumstances, employment, care or support provided, and health of the carer. In addition, carer participants will be asked to complete three standard tools: the Mayo-Portland Adaptability Inventory 4, WHOQOL BREF, and the Burden Scale for Family Caregivers.

### Instruments

***EQ-5D-3L.*** The EQ-5D-3L (3-level version) is a widely used measure of health-related quality of life ^31^. It comprises a descriptive system and a visual analogue scale. The descriptive system outlines five dimensions (5D) of health: mobility, self-care, usual activities, pain/discomfort and anxiety/depression ^31^. Respondents are asked to indicate what they are experiencing within 3-levels of difficulty (no problems, some problems, extreme, problems) represented numerically (1,2,3). The visual analogue scale displays a vertical measure from 0 to 100; where 0 is the ‘worst imaginable health’ and 100 is the ‘best imaginable health’ state. The respondent is asked to indicate the point on this scale that best represents their health state on the day of administration ^31^.

A description of the respondent’s health status is derived by combining the values related to the level of problems experienced in each dimension. For example, the health status of a person indicating no problems under mobility, some problems under self-care, some problems with usual activities, and extreme problems with both pain/discomfort and anxiety/depression, would be represented as ‘12233’. The numbers assigned to the level of problem under a dimension have no arithmetic properties ^31^.

Comparison of the EQ-5D-3L and the EQ-5D-5L (more precise measurement with the 5L), suggests that the EQ-5D-3L is prone to ceiling effects and may not accurately discriminate problems experienced at the mild level, therefore demonstrating ‘full health’ where mild problems exist ^32^. Other criticisms of both instruments are that they are not sufficiently sensitive in capturing psychological or social dimensions ^33^. However, the EQ-5D-3L is widely used and is considered a credible basis for clinical decision making ^34^. An advantage of the EQ-5D-3L over other measures for health-related quality of life (HRQoL), is its brevity, and therefore low burden for completion ^33^. Permission to use this tool in this study was obtained from the Euroqol Research Foundation.

***WHOQOL-BREF.*** The World Health Organisation (WHO) defines quality of life as an ‘individual’s perception of their life in the context of the culture and value systems in which they live and in relation to their goals, expectations, standards and concerns’ ^35^ (p.1). Quality of life is ‘a broad ranging concept which is affected in a complex way by the person’s physical health, psychological state, level of independence, social relationships, personal beliefs and their relationship to salient features of their environment’ ^36^ (p.1). The WHOQOL instruments may be used in particular cultural settings while allowing for cross-cultural comparisons ^35^. It can also be used to provide insight into the effect of disease on subjective well-being ^35^. The psychometric properties of the WHOQOL-BREF have demonstrated reliability and validity ^36, 37^. Although there is a lack of consensus on the suitability of this instrument in people with TBI, studies support the applicability of the WHOQOL-BREF in this population ^38, 39^. Permission to use the WHOQOL-BREF in this research was obtained from the World Health Organisation Press.

***European Brain Injury Questionnaire (EBIQ).*** The European brain injury questionnaire (EBIQ) was developed specifically for use with both brain injured patients and their relatives by Teasdale and colleagues ^40^. It comprises 63 questions regarding ‘problems or difficulties that people sometimes experience in their lives’. Respondents are requested to indicate if they have experienced these problems ‘not at all’, ‘a little’ or ‘a lot’, over the previous month. Items can be grouped into nine domains or scales: somatic, cognitive, motivation, impulsivity, depression, isolation, physical, communication and core, all of which demonstrated satisfactory levels of reliability with Cronbach’s α value of near or above 0.5 ^40^. Sopena and colleagues ^41^ found that the EBIQ demonstrated robust test-retest reliability for persons with brain injury and relatives of persons with brain injury; Schonberger and colleagues ^42^ report r values of 0.47–0.66,with all p values <0.001 for all scales. The EBIQ was designed for use with brain injury survivors and their relatives, on the basis that close relatives’ perspectives may balance lack of an awareness in the participant with TBI ^40, 41^. The EBIQ is freely available and specific permission is not required to use the instrument for research purposes.

***Mayo-Portland Adaptability Inventory 4.*** The MPAI-4 provides meaningful documentation of cognitive, behavioural and social challenges experienced by those who have acquired a brain injury. The MPAI-4 includes 30 items covering limitations commonly experienced by ABI survivors. Items are rated on a five-point scale from 0–4, where 0 represents ‘normal’ function and 4 ‘severe limitations’. The instrument also comprises three subscales: the ability index, adjustment index and the participation index. The MPAI-4 demonstrates good levels of clinical utility and psychometric quality, with very good construct validity and internal consistency ^43^ in people with TBI. A recent study in an Irish sample with ABI reported very good internal consistency for the total scale score (0.91) as well as the three subscales: abilities (0.94), adjustment (0.82) and participation indices (0.85) ^39^. The MPAI-4 is freely available and specific permission is not required to use the instrument for research purposes.

***Burden Scale for Family Caregivers (BSFC).*** The BSFC measures subjective burden in informal caregivers. It is available in 20 European languages, allowing for comparison between European populations ^44^. Subjective burden in those who provide care for the chronically ill has been found to significantly affect their emotional health, physical health and mortality as well as how the caregiver relates to the care receiver ^45^. The BSFC is a 28 item self-reporting instrument, that uses a four-point Likert scale ranging from ‘strongly agree’ to ‘strongly disagree’ ^45^. Split-half reliability test attained values of higher than 0.8 ^44^. The BSFC is freely available and specific permission is not required to use the instrument in research.

These instruments were chosen by experienced neuro researchers, to ascertain the quality of life, health states, effect of injury and difficulties experienced by the TBI survivor (EQ-5D-3L, WHOQOL-BREF, EBIQ). The MPAI-4 addresses cognitive, behavioural and social challenges. This data is essential to explore the state of health of TBI survivors in relation to the current state of neuro-rehabilitation in Ireland. The BSFC will provide date on the experiences of those who provide care or support the TBI survivor.

### Data management

Two research assistants will maintain a database on their encrypted, password protected computers. Hardcopy consent forms and survey responses will be stored separately and securely in locked cabinets in the offices of the researchers. Names and other contact details will be stored separately from completed questionnaires, whether on paper, or electronic format. Unique identifiers will be used by the DCU research team who will have sole access to the raw data and no identifiable information will be published. The DCU Risk and Compliance Officer has reviewed a personal data security schedule (PDSS) that lists the categories of personal data being processed. Data is available in the Open Science Framework data repository. On completion of the study, the archived dataset will be anonymised and lodged with the Irish Social Science Data Archive (ISSDA). If a TBI registry is established, anonymised data from this study will be shared with registry developers.

### Data analysis and statistical plan

An analysis of the data generated throughout the study will be reported to the Knowledge Users (Acquired Brain Injury Ireland and Headway). Consultation with a Patient and Public Involvement (PPI) advisory panel and a Research Advisory Group, set up as part of the wider team involved in this study, will also inform reports in collaboration with Knowledge Users and the research team.

Data points:

- Existing routing datasets
- Hospitals’ and voluntary organisations’ medical record data of TBI participants
- TBI participant surveys and six-month follow-up surveys
- Monthly accounts of the TBI survivors’ health care service usage for 6 months
- Carer survey data

Descriptive statistics using a range of univariate and multivariate statistical analyses will be employed to explore the data obtained through the partner sites and from participant surveys. Data will be collected and analysed using Qualtrics software ^46^, R ^47^ and SPSS ^48^.

### Reporting of results

The STROBE (STrengthening the Reporting of OBservational studies in Epidemiology) framework will be used ^49, 50^. We will report on eligibility; lack of participation, attrition numbers and reasons for same as well as those completing follow-up. The demographics, clinical condition on admission and discharge from acute care, rehabilitation and their use of health care on a continuum will be reported. Data from standardised instruments, participants’ financial situations, material changes and care circumstances will be presented.

### Bias

There are several potential sources of bias in this study. The first is that the criteria for entry into the study are imperfect as there are no uniform data collection systems for people with TBI in Ireland. While every effort will be made to identify people with moderate or severe brain injury correctly, and to exclude those with mild brain injury and those with very severe and profound brain injury, this is believed to be imperfect. There are no independent central sources of information on rehabilitation services that can be used to check reported use. To mitigate this, service use will be documented from participants prospectively, which should minimise error.

The potential for information bias exists, in particular because of the study participants. Respondents cognition may be less than optimal since injury, resulting in exaggeration or forgetfulness. The Covid-19 pandemic is another consideration where bias might occur as participants may not have been able to access rehabilitation or health care services because of lockdowns, resulting in alternative data compared to similar data collected under normal circumstances.

### Dissemination and knowledge translation

A formal knowledge translation plan has been designed in direct response to the needs of the TBI population identified by ABI Ireland and Headway. The findings will be applicable to these needs. The research protocol was developed in partnership with the researchers, Knowledge Users, the Public and Patient Involvement advisory panel and the Research Advisory Group.

The Knowledge Users are very experienced in managing political and policy advocacy campaigns and raising awareness of brain injury. The findings of this study will be directly applicable to these actions. In consultation with the PPI advisory panel, a plain language narrative synthesis of the research findings will be prepared and shared with key stakeholders. The research findings will also be shared with other organisations that find the data useful, for example, St. Doolagh’s Park Care and Rehabilitation Centre, Nua Healthcare, Redwood Extended Care Facility, The Irish Wheelchair Association, The Road Safety Authority and the Irish Medical Organisation. An open briefing will be held for members of the Irish Parliament (Teachta Dála), and senators in the Dáil.

The research team and the PPI panel will disseminate the final report. Members of the PPI panel are involved in this study to include the writing of the funding proposal. Advocacy efforts to influence health care pathways will be coordinated by ABI Ireland and Headway through the Neurological Alliance of Ireland (NAI). The NAI is instrumental in influencing health policy and practice on neuro-rehabilitation and has direct engagement with principal actors within the broader HSE clinical programme and the Department of Health. Knowledge Users, the researcher team, the Research Advisory Panel and the PPI panel will collaborate to propose solutions from the findings.

Knowledge Users and the PPI advisory panel have made a number of recommendations to disseminate the findings of this research study:

- A social media strategy, with partner organisations to disseminate the findings to people with brain injuries, their families and the wider public
- A launch seminar with all key stakeholders and other interested parties (for example, the Road Safety Authority, Irish Medical Organisation) to share findings
- A Policy Briefing Paper to outline the policy issues that arise from the research conference dissemination

Presentations at Irish, European, and international conferences. Manuscripts will be submitted to appropriate peer reviewed journals, such as Brain Injury, Neuro-epidemiology, The Journal of Head Trauma and Rehabilitation, and BMC Neurology (an open access, peer-reviewed journal).

There will be potential for further projects within the DCU/ Knowledge Users/ PPI partnership team, in particular around implementation of strategies, and the evaluation of interventions.

### Study status

This study is well underway with data collection complete and data analysis currently in progress. The expected completion data has been delayed because of barriers with data collection due to Covid-19. 

## Discussion

Advances in acute care have surpassed developments in rehabilitative care, resulting in increased demand for neuro-rehabilitation services ^11^, as more individuals who have experienced moderate or severe TBI are surviving. Increased demand, in turn, is contributing to longer waiting times for rehabilitation services, which are poorly configured to meet this demand ^11^. Previous research demonstrates that delayed rehabilitation can result in loss of function and unnecessary disability of TBI survivors ^11^, as well as pose significant challenges for their family members ^22^. The full scale of unmet needs in Ireland is unknown to date ^7, 20^, and rehabilitation pathways for this population are essentially undocumented. This study will address the current epidemiological data of TBI in Ireland and data on rehabilitation pathways for adult TBI survivors.

Ireland’s neuro-rehabilitation implementation plan outlines how rehabilitation services in Ireland might be reconfigured to achieve a flexible, responsive, accountable, rehabilitation service that can provide a standardised rehabilitation pathway ^11^. The service should be structured to deliver individualised rehabilitation locally, where possible, and in a timely and integrated manner, to meet the needs of service users ^11^. Through examining the rehabilitation pathways of individuals with moderate or severe TBI in Ireland, we expect that the current research findings will provide insight into the specific barriers to rehabilitation, and contribute valuable information to support the redevelopment of neuro-rehabilitation services. Additionally, acting on the knowledge of the current rehabilitation pathways has the potential to positively impact outcomes for TBI survivors currently navigating the system.

This study is the first in Ireland to examine how individuals use health care services following a TBI; it will provide a comprehensive view on health services usage and the rehabilitation services required of moderate or severe TBI survivors. The data derived from the study will help support efforts to maximise health service availability for TBI survivors locally and nationally. The research will explore the experiences of those providing care and support to an individual with TBI, many of whom are family members. Both international research and research within the Irish context demonstrate that is a considerable burden associated with providing care and support to TBI survivors ^20– 22^. Understanding the considerable role of informal carers in providing support to TBI survivors’ access to rehabilitation is of particular importance ^20^.

A dearth of research in the area of TBI in Ireland means that we do not fully understand the difficulties faced by individuals with moderate or severe TBI in accessing rehabilitation services. Health policy documents dating back to 2001 have acknowledged the need to develop rehabilitation services and, more recently, a specific focus on neuro-rehabilitation services has found that services are inadequate and poorly configured to meet demand ^11, 12^. A key area of challenge identified is the lack of reliable data on the TBI population ^9, 12^. In this context, the current study is timely in its focus on the epidemiology of TBI in Ireland and on rehabilitation pathways for TBI survivors. It is anticipated that findings from of this study will inform the aforementioned organisations, contribute to advocacy efforts for the redevelopment of neuro-rehabilitation services and make a much-needed contribution to the Irish literature on TBI.

## Data Availability

No data are associated with this article. Open Science Framework: Traumatic Brain Injury - Pathways to rehabilitation. https://doi.org/10.17605/OSF.IO/2BAUF ^29^. This project contains the following extended data: Carer-Family Member Questionnaire.pdf Participant materials HRB.pdf Person with TBI 1 ^st^ interview Questionnaire.pdf Person with TBI 2 ^nd^ Interview Questionnaire.pdf Person with TBI Health Care Usage.pdf Data are available under the terms of the Creative Commons Attribution 4.0 International license (CC-BY 4.0).

## References

[1] Centers for Disease Control and Prevention: National Center for Injury Prevention and Control. 2019. Reference Source

[2] Rubiano AM Carney N Chesnut R : Global neurotrauma research challenges and opportunities. *Nature.* 2015;527(7578):S193–S197. 10.1038/nature16035 26580327

[3] Popescu C Anghelescu A Daia C : Actual data on epidemiological evolution and prevention endeavours regarding traumatic brain injury. *J Med Life.* 2015;8(3):272–277. 26351526 PMC4556905

[4] Brazinova A : Epidemiology of Traumatic Brain Injury in Europe: A Living Systematic Review. *J Neurotrauma.* 2015. 10.1089/neu.2015.4126 PMC8082737 26537996

[5] Peeters W van den Brande R Polinder S : Epidemiology of traumatic brain injury in Europe. *Acta Neurochir (Wien).* 2015;157(10):1683–1696. 10.1007/s00701-015-2512-7 26269030 PMC4569652

[6] Tenant A : Acquired Brain Injury: The Numbers Behind the Hidden Disability. 2018;9. Reference Source

[7] Phillips J : National Report on Traumatic Brain Injury in the Republic of Ireland 2008. 2008;67. Reference Source

[8] Staines A O’Farrell A Healy A : Accuracy of identification of traumatic brain injuries in routine hospital data. *Euro J Pub Health.* 2021;31(3). 10.1093/eurpub/ckab165.642

[9] McGettrick G : National Policy and Strategy for the Provision of Neuro-Rehabilitation Services in Ireland 2011 – 2015.Policy Briefing Paper No. 2. 2015.

[10] Jourdan C Bayen E Bosserelle V : Referral to rehabilitation after severe traumatic brain injury: results from the PariS-TBI Study. *Neurorehabil Neural Repair.* 2013;27(1):35–44. 10.1177/1545968312440744 22460612

[11] Health Services Executive: National Strategy and Policy for the Provision of Neuro-rehabilitation Services in Ireland. From Theory to Action. Implementation framework. 2019;96. Reference Source

[12] Burke S McGettrick G Foley K : The 2019 neuro-rehabilitation implementation framework in Ireland: Challenges for implementation and the implications for people with brain injuries. *Health Policy.* 2020;124(3):225–230. 10.1016/j.healthpol.2019.12.018 31964508

[13] European Physical and Rehabilitation Medicine Bodies Alliance: White book on physical and rehabilitation Medicine (prM) in Europe. Chapter 5. The prM organizations in Europe: structure and activities. *Eur J Phys Rehabil Med.* 2018;54(2):198–213. 10.23736/S1973-9087.18.05149-3 29565106

[14] Mitchell E Ahern E Saha S : Neuropsychological rehabilitation interventions for people with an acquired brain injury. A protocol for a systematic review of economic evaluation [version 2; peer review: 2 approved]. *HRB Open Res.* 2020;3:83. 10.12688/hrbopenres.13144.2 33367203 PMC7684672

[15] Turner-Stokes L Dzingina M Shavelle R : Estimated Life-Time Savings in the Cost of Ongoing Care Following Specialist Rehabilitation for Severe Traumatic Brain Injury in the United Kingdom. *J Head Trauma Rehabil.* 2019;34(4):205–214. 10.1097/HTR.0000000000000473 30801440 PMC6687405

[16] Deasy C Cronin M Cahill F : Implementing Major Trauma Audit in Ireland. *Injury.* 2016;47(1):166–172. 10.1016/j.injury.2015.07.016 26315666

[17] Sigurdardottir S Andelic N Wehling E : Return to work after severe traumatic brain injury: a national study with a one-year follow-up of neurocognitive and behavioural outcomes. *Neuro Rehab.* 2020;30(2):281–297. 10.1080/09602011.2018.1462719 29667477

[18] World Health Organization: Health Inequities and their causes.2018. Reference Source

[19] Copley A McAllister L Wilson L : We Finally Learnt to Demand: Consumers’ Access to Rehabilitation Following Traumatic Brain Injury. *Brain Impair.* 2013;14:436–449. 10.1017/BrImp.2013.32

[20] Graff HJ Christensen U Poulsen I : Patient perspectives on navigating the field of traumatic brain injury rehabilitation: a qualitative thematic analysis. *Disabil Rehabil.* 2018;40(8):926–934. 10.1080/09638288.2017.1280542 28129694

[21] McDermott GL McDonnell AM : Acquired brain injury services in the Republic of Ireland: Experiences and perceptions of families and professionals. *Brain Inj.* 2014;28(1):81–91. 10.3109/02699052.2013.857790 24328803

[22] Muldoon OT Walsh RS Curtin M : Getting my life reset Living with an acquired brain injury: The Irish experience. 2017. Reference Source

[23] Turner-Stokes L Pick A Nair A : Multi-disciplinary rehabilitation for acquired brain injury in adults of working age. *Cochrane Database Syst Rev.* 2015; (12):CD004170. 10.1002/14651858.CD004170.pub3 26694853 PMC8629646

[24] British Society of Rehabilitation Medicine: Rehabilitation for patients in the acute care pathway following severe disabling illness or injury: BSRM core standardsfor specialist rehabilitation. 2014;23. Reference Source

[25] Scarpino M Lanzo G Hakiki B : Acquired brain injuries: neurophysiology in early prognosis and rehabilitation pathway. *Signa Vitae.* 2021;17(5):1–10. 10.22514/sv.2021.132

[26] Department of Health: National Policy and Strategy for the Provision of Neuro-rehabilitation services. 2011;139. Reference Source

[27] O’Neil ME Carlson K Storzbach D : Complications of Mild Traumatic Brain Injury in Veterans and Military Personnel: A Systematic Review.2013. 24600749

[28] Assisted Decision-Making (Capacity) Act 2015.(Ireland) PT1. S.2, 3(2). Reference Source

[29] Staines A Corrigan C O'Donnell K : Traumatic Brain Injury - Pathways to rehabilitation.2021. 10.17605/OSF.IO/2BAUF

[30] Data Protection Commission: Guidance Notes: Guidance on anonymisation and pseudoanonymisation.2019. Reference Source

[31] The EuroQol Group: EuroQol--a new facility for the measurement of health-related quality of life. *Health Policy.* 1990;16(3):199–208. 10.1016/0168-8510(90)90421-9 10109801

[32] Thompson AJ Turner AJ : A Comparison of the EQ-5D-3L and EQ-5D-5L. *Pharmacoeconomics.* 2020;38(6):575–591. 10.1007/s40273-020-00893-8 32180162

[33] Geraerds AJLM Bonsel GJ Janssen MF : The added value of the EQ-5D with a cognition dimension in injury patients with and without traumatic brain injury. *Qual Life Res.* 2019;28(7):1931–1939. 10.1007/s11136-019-02144-6 30820809 PMC6571097

[34] Berger-Estilita J Granja C Gonçalves H : A new global health outcome score after trauma (GHOST) for disability, cognitive impairment, and health-related quality of life: data from a prospective cross-sectional observational study. *Brain Inj.* 2019;33(7):922–931. 10.1080/02699052.2019.1581257 30810390

[35] World Health Organization Division of Mental Health and Prevention of Substance Abuse: WHOQOL : measuring quality of life.12. Reference Source

[36] Skevington SM Lotfy M O’Connell KA : The World Health Organization's WHOQOL-BREF quality of life assessment: psychometric properties and results of the international field trial. A report from the WHOQOL group. *Qual Life Res.* 2004;13(2):299–310. 10.1023/B:QURE.0000018486.91360.00 15085902

[37] Polinder S Haagsma JA van Klaveren D : Health-related quality of life after TBI: a systematic review of study design, instruments, measurement properties, and outcome. *Popul Health Metr.* 2015;13:4. 10.1186/s12963-015-0037-1 25722656 PMC4342191

[38] Chiu WT Huang SJ Hwang HF : Use of the WHOQOL-BREF for Evaluating Persons with Traumatic Brain Injury. *J Neurotrauma.* 2006;23(11):1609–1620. 10.1089/neu.2006.23.1609 17115908

[39] Fortune DG Walsh RS Waldron B : Changes in aspects of social functioning depend upon prior changes in neurodisability in people with acquired brain injury undergoing post-acute neurorehabilitation. *Front Psychol.* 2015;6:1368. 10.3389/fpsyg.2015.01368 26441744 PMC4561758

[40] Teasdale TW Christensen AL Willmes K : Subjective experience in brain-injured patients and their close relatives: a European Brain Injury Questionnaire study. *Brain Inj.* 1997;11(8):543–563. 10.1080/026990597123250 9251864

[41] Sopena S Dewar BK Nannery R : The European Brain Injury Questionnaire (EBIQ) as a reliable outcome measure for use with people with brain injury. *Brain Inj.* 2007;21(10):1063–1068. 10.1080/02699050701630342 17891569

[42] Schönberger M Humle F Teasdale TW : Subjective outcome of brain injury rehabilitation in relation to the therapeutic working alliance, client compliance and awareness. *Brain Inj.* 2006;20(12):1271–1282. 10.1080/02699050601049395 17132550

[43] Malec JF Kean J Altman IM : Mayo-Portland Adaptability Inventory: Comparing Psychometrics in Cerebrovascular Accident to Traumatic Brain Injury. *Arch Phys Med Rehabil.* 2012;93(12):2271–2275. 10.1016/j.apmr.2012.06.013 22743410

[44] Gräsel E : Somatic symptoms and caregiving strain among family caregivers of older patients with progressive nursing needs. *Arch Gerontol Geriatr.* 1995;21(3):253–266. 10.1016/0167-4943(95)00660-d 15374201

[45] Gräsel E Chiu T Oliver R : Development and Validation of the Burden Scale for Family Caregivers.2003;25. Reference Source

[46] Qualtrics software, Qualtrics. Provo, UT, USA. Reference Source

[47] R Core Team: R: A language and environment for statistical computing.R Foundation for Statistical Computing.2019. Reference Source

[48] IBM Corp: IBM SPSS Statistics for Windows.Version 27.0. Armonk, NY: IBM Corp. Released2020.

[49] Lachat C Hawwash D Ocké MC : Strengthening the Reporting of Observational Studies in Epidemiology-Nutritional Epidemiology (STROBE-nut): An Extension of the STROBE Statement. *PLoS Med.* 2016;13(6):e1002036. 10.1371/journal.pmed.1002036 27270749 PMC4896435

[50] University of Bern: STROBE Statement. Strengthening the reporting of observational studies in epidemiology.2009. Reference Source

