# Supplementary material for: Traumatic brain injury epidemiology and rehabilitation in Ireland: a protocol paper
Source: HRB Open Res. 2022 Sep 22;4:66. Originally published 2021 Jun 15. [Version 2] doi: 10.12688/hrbopenres.13209.2 (PMC10579856; doi:10.12688/hrbopenres.13209.2)
Supplement: Peer review history

# Reviewer report: Reviewer response for version 2

**DOI:** 10.21956/hrbopenres.14875.r35187

**Published:** 2023-10-16

**Authors:** Katrin Rauen

**Affiliations:** 1 University of Zurich, Zurich, Switzerland

Dear authors, thank you very much for your revision of your protocol. There is no doubt that TBI research is necessary in Ireland. I suggest to adopt your protocol for comparability as follows:

1. Please double check your clear aims 1-3 from the abstract and repeat those in the main text. In the current status there is a discrepancy, and thus the protocol would benefit from revising this section.
2. Why did the quthors did not incorporate the CENTER-TBI recommendation for assessing outcome with the current gold standard (GOSE) and generic (SF-36v2 or SF-12v2) and disease-specific HRQoL (QOLIBRI instrument). Please compare for TBI outcome assessments the citation below:
https://www.center-tbi.eu/project/validated-translations-outcome-instruments
3. It would be of major interest how many TBI patients received in- and out-patient neurorehabiltion
4. It would be of interest to assess time from TBI to admission to neurorehabilitation.
5. Citing relevant TBI outcome publications on HRQoL would be beneficial.

Is the study design appropriate for the research question?

Partly

Is the rationale for, and objectives of, the study clearly described?

Partly

Are sufficient details of the methods provided to allow replication by others?

Partly

Are the datasets clearly presented in a useable and accessible format?

Partly

Reviewer Expertise:

Neurotrauma, Traumatic brain injury, HRQoL, neuropsychiatric outcome after TBI, sleep disorders, depression, sex- and gender-specific depression, post-concussion syndrome

I confirm that I have read this submission and believe that I have an appropriate level of expertise to confirm that it is of an acceptable scientific standard, however I have significant reservations, as outlined above.

# Reviewer report: Reviewer response for version 2

**DOI:** 10.21956/hrbopenres.14875.r32908

**Published:** 2022-10-03

**Authors:** Dominic Trepel

**Affiliations:** 1 School of Medicine, Trinity College Dublin, Dublin, Ireland; 2 Global Health Health Institute (TCD | UCSF), San Francisco, CA, USA

Thank you for considering the reviewers' suggestions and for the pithy rebuttals. Having reviewed the manuscript, comments on the protocol have been addressed as best possible (given that the project is now completed) and the work should be indexed.

Is the study design appropriate for the research question?

Partly

Is the rationale for, and objectives of, the study clearly described?

Partly

Are sufficient details of the methods provided to allow replication by others?

No

Are the datasets clearly presented in a useable and accessible format?

Partly

Reviewer Expertise:

Health economics

I confirm that I have read this submission and believe that I have an appropriate level of expertise to confirm that it is of an acceptable scientific standard.

# Reviewer report: Reviewer response for version 1

**DOI:** 10.21956/hrbopenres.14361.r29933

**Published:** 2021-09-07

**Authors:** Dominic Trepel

**Affiliations:** 1 School of Medicine, Trinity College Dublin, Dublin, Ireland; 2 Global Health Health Institute (TCD | UCSF), San Francisco, CA, USA

**Summary**:

 Thanks you for inviting input to this intended work, it is admirable that the research group outlines their intentions in this open peer review process at the protocol stage.

 The work is very much needed to improve the current state of research on rehabilitation services for brain injuries in Ireland and this draft provides a good summary of the intentions. However, in outlining multiple aims, the intended study became a little confusing to read and often less than clearly described. It is recommended one aim is stated (e.g. to establish a prospective cohort of survivor of a brain injury) and then state the research questions this will be answered (e.g. estimate prevalence/incidence). It might also be beneficial to present other questions in separate protocol papers as this would allow sufficient methodological detail to allow replication. Despite these methodological issues, overall this protocol is well received and sets out an exciting agenda.

 Here are specific comments the paper should address (presented by section):

**TITLE**:

 Specify the methods in the title (e.g. a protocol "for a prospective cohort study")

**ABSTRACT**

 Background:

 Move sentence in Aims (i.e."This is the protocol for a ["mixed methods" OR perhaps more accurately "prospective cohort"] study that will examine pathways through rehabilitation for survivors of TBI in the Republic of Ireland") to be last sentence of background.

**Aims**:

 Clearly state Aims numerically:

 Aim(s):

 1. To explore the experiences of family members providing care or support to a person with TBI;

 2. To estimate the incidence and prevalence of TBI in Ireland.

 [But having read the paper, it was difficult to be clear on the aim and became confused if it was actually create a 'prospective cohort study'. Also, I am struggling to find relationship to "experiences of family members" (which was assumed indicated additional qualitative methods).]

 Final sentence in Aims may not be a research aim. Considering as a aim if could be stated as:

 3. To support advocacy efforts by providing epidemiological data and information on how people with TBI access rehabilitation and health services

 ... however, 'advocacy' is not research aim (i.e that you will specify a research method to achieve) but could rather be more a considered a conflict of interest. Consider either removing. Or, if the aim is integral to this proposed research, maybe consider reworking to indicate a 'translational research' aim.

 Cross reference to ensure that aim(s) stated in body text are aligned with those stated at abstract level, and that any stated aim clearly links to the related methodology.

 Also, as stated above, the protocol paper might be easier to follow describe one aim (e.g. the prevalence study) and other aims (e.g. qualitative study, or, assessment of resource use) might better be presented in a separate Protocol paper. This would allow more clear elaboration in describing methods (see below for more details).

**Method**:

 Clearly describe methods that align with stated aims (above). For example, it is unclear to which stated aim the statement: "Questionnaires will be administered to participants with TBI on two separate occasions, six months apart, and to family members providing care or support to an individual with TBI, on one occasion."

 relates -  is this part of qualitative exploration or quantifying prevalence/incidence?

 clarify statement "medical records of participants will be abstracted" - do you mean data will be 'extracted' from medical record to create a dataset? And, if so, be more explicit on what is "key information" and provide explicit detail on the process from identifiable patient record to a dataset fit for analysis that conforms with GDPR . As it currently stands, this method is vague and unclear how it relates to the aim.

 It is unclear how the methods relates to stated aims: "TBI survivor participants’ use of health care will be followed prospectively for six months."

**Expected outcomes**:

 Following statement suggest a new and additional aim (ie which is different to those stated above): "pathways through rehabilitation in Ireland, to understand how rehabilitation services are accessed". If this is related to qualitative interviewing, try to make more congruent and then indicate methodologically 'how' an expected outcome will be delivered (e.g. a grounded theory that indicates the pathway (etc.) and then adjust *Aim 1* to not solely reflect care partner(s) experience)

**INTRODUCTION**

 Rubiano et al 2015 does not support statement that TBI 'is a leading cause of death and disability worldwide".

 Statement " *In Ireland the incidence and prevalence of TBI is unknown*" is central, as is *" there is no national mechanism for “capturing the incidence, management and outcome of TBI presenting” to the health care system". *However, to the latter statement greater elaboration on WHY 'incidence, management and outcomes" are such an unknown in Ireland would be helpful to a more international audience (e.g. for example, is is because both US and UK estimates benefit from more solid routinely collected administrative data, or because Ireland does not have unique patient identifiers?)

 A clear and early definition of what is considered to constitute "rehabilitation' may be useful. Currently, the protocol makes an immediate assumption all readers should be aware what might constitute a "rehabilitation pathway" and also that rehabilitation does not vary.

 Change statement "...intensive and early access to neuro-rehabilitation **[may be]** cost-effective ^9^^,^^10^" (NB neither referenced papers by McGregor (1997) or Turner-Stokes (2019) are evidence ono cost-effectiveness) -  consider referring the forthcoming review [1] which does more comprehensively support the assertions on cost effectiveness of rehabilitation for brain injury

 RE  - with respect to reference to Turner-Stokes editorial in 2004, this is not evidence on cost effectiveness but rather shows "long- term cost savings would outweigh short-term rehabilitation costs in a UK setting for those with serious brain injuries" (i.e. provides an opinion that rehabilitation is cost-minimising) -  Again, recommend reference to Mitchell et al work which provides a critical appraisal of the health economic evidence base ^1^.

 Query statement "estimated that 40% of brain injury survivors will have a moderate to severe disability". Does this assume that incidence of brain injury (ie. denominator of total brain injuries) was established by Philips? Given aims outlined in this protocol (ie. incidence), perhaps statement could be more tenuous. 

 "As is the case internationally, difficulty navigating the health system ^17^^,^^20^ and poorly configured, inefficient, funding streams have been shown to relate to these gaps and delays". Some caution is required in terms of the rigour of supporting evidence. For example, HSE report is more of an action plan rather than a source of evidence and therefore assertions (e.g. level of relative efficiency) may not grounded in fact (i.e. have academic rigour).

 Statement (and subsequent paragraph)"It is expected that the findings of this research study will contribute to the literature on TBI in Ireland in a number of ways" is indicative  that the protocol paper is too broad and may merits greater focus to explain any one well. Providing robust estimates of population level prevalence/incidence of TBIs in Ireland would be a solid and welcome contribution. The description of pathway through services (which I assume is qualitative in nature), experience of carers (not to mention more general resource use measurement) and formulation of knowledge translation plan are all also good aims but surely benefit separate protocol papers (so to allow sufficient detail for replication).

**PROTOCOL** 

**Ethical approva**l:

 Attach ethics submission DCUREC/2018/123 as a supplement to this Protocol paper.

**Primary and secondary aims:**

 As indicated above, recommend selection one primary aim:

 RE "To describe the incidence, prevalence and patterns of disability associated with moderate to severe TBI survivors" - Reading further, as this is not a population prevalence, it is not (as stated in abstract) aiming to "estimate the incidence and prevalence of TBI in Ireland" but rather (I assume) prevalence of subtypes of TBIs.

 I am also confused by aim on 'incidence' which would introduce temporal aspect to the study (i.e. rates of TBI going up or down over time). Over time, it might be of interest (and feasible) to explore relationships between incidence of disability types, service use and/or demand for informal care (e.g. by exploiting the 6-month follow up), but again, this could validly constitute a completely separate paper (although, it would be commendable of the formulated datasets were designed to allow such future questions to be answered).

 Statement of aim to improve "knowledge of rehabilitation pathways for TBI survivors" is vague in terms of what it is aiming to show

**Study design**

 Statement "This is a quantitative, descriptive cohort study involving survivors of moderate to severe TBI " suggest that the primary aim is qualitative, so perhaps consider indicate qualitative part of mixed methods as a secondary aim (also study design makes not references to the qualitative design as indicated in the abstract reference to "mixed method").

**Sampling plan**

 Again, with earlier reference to "prevalence in Ireland", the limitation of sampling from hospitals only needs to clearly acknowledged throughout.

**Sample size calculation**

 Clarify: What is the sample size detecting? This should be related to your primary aim (and an associated hypothesis test?)

**Inclusion criteria:**

 Criteria for level of severity is a little confusing do participants need to have all three, or at least one of the criteria. Currently, I interpret as just one and, if that is correct, recommend rephrasing to "must satisfy at least one of the following" (NB: exclusion that "TBI survivors with mild trauma (classified by GCS >12)" should also indicate similar of LOC and PTA to exclude)

 Elaborate on protocol for: "If these measures of injury severity are not available, positive findings on computerised tomography (CT) or magnetic resonance imaging (MRI) will be used to determine injury severity." May require cross reference to a  supplement, if extensive?

 Consider changing "family member" to "care partner" (or "non-professional care partner" to be exact) as: 1. assumes occurs in diads and; 2. refers to non-profession caregiver" (which may not be family). 

**Data collection**

 The "Extended data ^26^^" ^is well received and therein the "Questionnaires and materials" have been inspected. However, content of suppository is greater than just  and might better directly point reader to specific hyperlink for the "letter of invitation, a patient information leaflet and a patient consent form" (https://osf.io/nhbt8/). Authors need to better orientate read through these materials.

 "Extended data" also provides three questionnaire to person with TBI and one for carer, and the deployment process for these could be explained (e.g. I note reference to "Qualtrics Survey Software" which could be explained)

 - The survey questionnaires seem to expect quite a time commitment from participants, which raise concerns that, overall, data completeness will suffer (particularly when participants are asked at follow up timepoints to contribute their time again). Has the survey been piloted ahead of moving to collecting data from n=200? If so, to relay concerns that research does not overly burden on survey respondents, please  report average time to complete survey (available from survey tool) for each of the following materials:

 Carer_Family Member Questionnaire (70 pages - Ref:  https://osf.io/bxjnw/)

 Person with TBI 1st interview Questionnaire (36 pages -Ref: https://osf.io/n46ea/ )

 Person with TBI 2nd Interview Questionnaire (30 pages - Ref:  https://osf.io/53hx9/)

 - Person with TBI Health Care Usage (5 pages -  Ref: https://osf.io/5cjpz/)

 - furthermore, outcomes make reference to "EQ-5D-3L ^28^, WHOQOL BREF ^29^, European Brain Injury questionnaire (EBIQ) ^30^ "

 RE "Surveys will be administered to all participants" -  It should be assumed that some for people with severe TBI may have impaired ability to complete surveys, clarification required on what is the process will be in such situations (e.g. proxy report?)

 Under " **Instruments**"

 The addition of the quality of life measure such at EQ5D3L and WHOQOL, whilst welcomed, do not appear linked to any of the stated aims. Also, it is unclear what they contribute  (recommend consulting with a trained health economist  on quality of life and also for developing robustly designed ' *bespoke'* resource use measurement tools).

 Consider that the MPAI-4 is routinely collected in brain injury and therefore collecting primary data may add unduly to respondent burden.

 The study appears to primarily be developing a 'prospective cohort' (with a good deal of rich data for each individual and carer) and this should be reflected under **Data analysis and statistical plan. **As part of outlining this plan, it would be very helpful if the authors would comment to providing empty tables to indicate what descriptive statistics will be reported in the final paper. Also, with respect to analysis (i.e. "univariate and multivariate statistical analyses ") further detail should be provided to explain what outputs this aims to provide (ie to answer salient research questions). 

**Bias**

 There are likely many more sources of bias that may, and should, be considered ahead of rolling out this research plan.

 It would be helpful if the team could commit to documenting and reporting consideration of potential sources of bias and committing to conducting postdoc analyses to determine whether they are valid concerns.

**Dissemination and knowledge translation**

 Strong dissemination plan with good involvement of PPI. One recommendation is to commit to seek peer review of outputs by people living with brain injuries (In terms of grant funding, there is likely a PPI review panel at funding application stage).

**DISCUSSION**

 Reference to "increasing demand" and "poor configuration to meet demand" sets a policy agenda of HSE. However, it would be helpful if the group could ensure that economic aspects such as "supply" and "demand" are considered in design of the datasets -  highly recommend the research group collaborating with a health economist. 

 The study purports to be the first study in Ireland to *"examine how individuals use healthcare services following a TB*I". However, having considered the study design and inspected the questionnaire design, the study is not population-based and focuses on survivors  (so will not accurately reflect all service use). Also the "bespoke" resource questionnaire may likely have issues as may result in missing data due to not considering zero, and operationalising methods to deal with zero service users. Finally, this seems a deviation from the stated aims

 Throughout the paper, there are several reference to equity of access (as it also mentioned in the discussion) however, equity (or indeed inequality) has not been defined anywhere to be operationalised and further detail on data collected and the associated analysis plan would be required.

**Data availability**

**Underlying data: **it is highly recommended that the study be initially piloted in a small number and some preliminary data be provided

**Extended data: **well received however further orientation to each questionnaire  (and their merits) would be welcomed. At present, there data collection appears so extensive that, overall, overburdening respondence may compromise overall quality of the study. 

**Acknowledgments**

 Specify sources of funding which were received, providing grant numbers etc.

Is the study design appropriate for the research question?

Partly

Is the rationale for, and objectives of, the study clearly described?

Partly

Are sufficient details of the methods provided to allow replication by others?

No

Are the datasets clearly presented in a useable and accessible format?

Partly

Reviewer Expertise:

Health economics

I confirm that I have read this submission and believe that I have an appropriate level of expertise to confirm that it is of an acceptable scientific standard, however I have significant reservations, as outlined above.

## References

[1] : Neuropsychological rehabilitation interventions for people with an acquired brain injury. A protocol for a systematic review of economic evaluation. *HRB Open Res* .2020;3: 10.12688/hrbopenres.13144.2 83 10.12688/hrbopenres.13144.2 33367203PMC7684672

# Response

**Published:** 2022-08-17

**Authors:** Catherine Corrigan

**Affiliations:** Dublin City University, Ireland

Thank you for your time to provide this valuable review. We have taken the steps to address each issue.

**TITLE** modified to reflect cohort study; it is not entirely prospective however, therefore 'prospective' was not added.

**ABSTRACT**

**Background** – last sentences added to background as recommended.

**Aims **are now clearly stated numerically. The abstract aims align with the aims in the body of the paper and link to the methodology. As this is an extensive study, substantial outcomes are expected. Thank you for the suggestion for separate protocol papers, however as the study is ending, we are focusing on reporting outcomes at this stage.

**Method** – additional information added to this section in the abstract to reflect the use of existing routine data as epidemiological sources. Key information is expounded on. See GDPR details under ‘data collection’ and ‘materials’ headings. Clarification for the 6-month follow-up re participants' use of health and rehabilitation services has been added.

**Expected outcomes **are clarified to align better with the aims of the study.

**INTRODUCTION**

 Rubiano et al. article indicate ‘within the spectrum of trauma related injuries, TBI and SCI are the largest causes of death and disability’ – sentence modified to reflect trauma related.

 Incidence and prevalence of TBI in Ireland is unknown - no unique ICD-10 code exists for TBI.

 An explanation of rehabilitation is provided in the introduction with sources cited.

 RE: “ *neither referenced papers by McGregor (1997) or Turner-Stokes (2019) are evidence onto cost-effectiveness…”* McGregor is removed; however in the Turner Stokes (2019) study, findings indicate: “specialist rehabilitation proved highly cost-efficient for patients severely disabled by severe TBI ”. Thank you for the Mitchell et al. source now cited.

 RE: “ *T* *urner-Stokes editorial in 2004, this is not evidence on cost effectiveness...” *The author indicates: “there is an increasingly strong evidence base for the effectiveness and cost-effectiveness of rehabilitation following acquired brain injury’’; however, we appreciate the update from Mitchell et al. (added) indicating the lack of evidence of cost-effectiveness of neuropsychological rehabilitation.

 RE: *"estimated that 40% of brain injury survivors will have a moderate to severe disability"*

 This incidence was established by the Traumatic Coma Databank; we were unable to locate the original source, nor find an updated source with that information, therefore the reference from the Phillips report had not been changed.

 RE: *"As is the case internationally, difficulty navigating the health system ^.^...”*

 While we appreciate your comment on the rigour of the supporting evidence, HSE indicate that their document is a “scientific approach based on data”. Additionally, we are aware of this work as Dr Jacinta McElligott (co-chair of the National Strategy Group on the HSE document) is a co-applicant and we have met with the HSE regarding the mapping of neuro-rehabilitation services in Ireland. 

 RE: *“* *It is expected that the findings of this research study will contribute to the literature on TBI in Ireland in a number of ways"… *As above, re separate protocol papers: the study is ending and we are focusing on reporting outcomes at this stage. This is an extensive study generously funded by HRB, ABII and Headway, that merits substantial outcomes.

**PROTOCOL**

**Ethical approval:**

 RE: *“Attach ethics submission…”*

 Attaching the ethics submissions is not a requirement.

**Primary and secondary aims:**

 As above, this is an extensive study generously funded by HRB, ABII and Headway, that merits substantial outcomes with more than one primary aim.

 RE: *"To describe the incidence, prevalence and patterns of disability associated with moderate to severe TBI survivors"… *‘incidence and prevalence’ has been replaced with ‘epidemiological data’ in the context of the aims of the study. Existing routine datasets such as the Major Trauma Audit has been added to the manuscript and will assist with the epidemiological data for this study.

 RE: *“* *I am also confused by aim on 'incidence' which would introduce temporal aspect to the study”…*

 Incidence is the occurrence of new cases over a specific time period which we had hoped to capture from the cohort that sustained brain injury within 3-12 months; however, our recruitment of new cases (Cohort 1) was poor and we will likely not report on estimated incidence rates. The dataset is extensive and will be available for other papers on the Open Science framework.

 RE: *"knowledge of rehabilitation pathways for TBI survivors"… *the wording of this aim has been modified to read ‘document the rehabilitation experiences of TBI survivors in Ireland’.

**Study design**

 RE: *"This is a quantitative, descriptive cohort study involving survivors of moderate to severe TBI "*…

 The primary focus of the study is the epidemiological data; however, some qualitative data will likely be derived from monthly accounts of current health service usage, which we will report if deemed useful in response to the study objectives. We removed the mixed method error.

**Sampling plan**

 RE: *“* *Again, with earlier reference to "prevalence in Ireland", the limitation of sampling *from hospitals only”… The “current study is undertaken in partnership between two leading Irish brain injury organisations, Acquired Brain Injury Ireland and Headway…” and is not limited to hospitals only.

**Sample size calculation**

 RE: *“what is the sample size detecting? *Epidemiological data on TBI in Ireland.

**Inclusion criteria:**

 RE: *“* *Criteria for level of severity is a little confusing…” *Level of severity is modified to moderate *or* severe throughout the revised manuscript. LOC and PTA are added to the mild TBI (exclusion criteria). CT, MRI results etc. added – thank you for this as we did extract that data from the medical records.

 RE: *“Consider changing family members...” *Family members now changed to 'carers'.

**Data collection**

 RE: *“The “Extended Data” is well received and therein…better directly point reader to specific hyperlink”*… We will take your recommendations on board and revisit the content in OSF.

 RE: *“Extended data” also provides three questionnaires…” *Under Study design: “Surveys will be completed, a) in person in a suitable location proposed by the participants, b) over the phone, or c) online. Deployment process added. The Qualtrics reference is added.

 RE: *“The survey questionnaires seem to expect quite a time commitment…has the study been piloted…” *Yes, we piloted the study with 5 TBI survivors. ABII and Headway were consulted regarding the length of the questionnaire and the potential for participant burden; members from the PPI panel reviewed the questionnaire as well; this resulted in a shorter questionnaire; the research assistants are experienced in communicating with brain injury survivors and are very sensitive to fatigue of difficulties with attention of the participants; participants can stop during the survey and take breaks to rest; survivors were willing participants and sometimes completed the survey over two sittings or more.

 RE: *“Carer_family member questionnaire…report average times to complete the survey”*

 We used Qualtrics for the carer_family member questionnaire. A participant could start and stop the survey at intervals, meaning complete it over days; there was no pressure to complete it within a specified time limit.

 RE: *"Surveys will be administered to all participants"…*

 “individuals who have capacity to give informed consent” is an inclusion criterion which excludes people with impaired ability. Capacity to give informed consent is also detailed. Proxy data is not permitted.

**Instruments**

 RE: *“The addition of the quality of life measure such as EQ5D3L and WHOQOL…” *Additional information on the instruments to indicate alignment with stated aims has been provided.

 RE: *“Also it is unclear what they contribute…” *Thank you for the recommendation to consult with a health economist which we will take on board.

 RE: *“Consider that the MPAI-4 is routinely collected in brain injury*…” Please refer to the above response on how the burden on respondents was addressed. Additionally, data retrieved from the MPAI-4 that is commonly used in the field of brain injury studies allows other researchers to compare findings.

 RE: *“The study appears to primarily be developing a ‘prospective cohort’…” *The study has two cohorts and is not considered primarily prospective. Populated tables will be provided when the data is ready for analysis. Details of data analysis in relation to the RQs and the aims of the study will be provided in a separate outputs paper.

**Bias**

 RE: *“There are likely many more sources of bias…” *Information bias and potential bias as a result of the Covid-19 pandemic was added. Thank you for the post doc analysis suggestion.

**Dissemination and knowledge translation**

 RE: *“Strong dissemination plan…” *A PPI panel is involved in this study to include the writing of the proposal for funding.

**DISCUSSION**

 RE: *“Reference to “increasing demand” and “poor configuration to meet demand” sets a policy…” *Thank you again for the recommendation to consult with a health economist which we will take on board.

 RE: *“The study purports to be the first study in Ireland…” *We appreciate that the study will not accurately reflect all service use and that is not the intention, rather we want to learn accessibility and appropriateness of services being uses by the TBI survivor. This relates to the aims where TBI survivors may be using, have access to or not, services not appropriate for the best chance of optimizing their quality of life, e.g. frequent GP visits c/o pain when adequate mobility/exercise regime could mitigate this complaint.

 RE: *“Throughout the paper, there are several references to equity of access…”*

 Inequity is defined (WHO) and equity has been removed from the discussion section as per your recommendation.

**Data availability**

 RE: Underlying data: ***“*** *it is highly recommended that the study be initially piloted…”*

 We piloted the study with 5 TBI participants and modified accordingly.

 RE: Extended data: ***“*** *The extended data is* *well received however further orientation to each questionnaire…” *Reasons for the choice of instruments (by experienced neuro researchers) are provided. We appreciate the extensive data collection and have closely considered the potential burden on the respondents. Both research assistants have experience in working with people with brain injury and are well educated on brain injury awareness. Respondents will be assisted to complete the questionnaire and will be informed they can take breaks to rest.

 RE: *“Extended data also provides three questionnaires…” *The use of the questionnaires and the deployment process are explained.

 Thank you.

# Reviewer report: Reviewer response for version 1

**DOI:** 10.21956/hrbopenres.14361.r29835

**Published:** 2021-08-12

**Authors:** Laraine Winter

**Affiliations:** 1 Villanova University, Villanova, PA, USA; 2 Philadelphia Research and Education Foundation, Corporal Michael J. Crescenz Veterans Affairs Medical Center, Philadelphia, PA, USA

This ms describes the protocol for an on-going study that will examine incidence and prevalence of moderate to severe TBI in Ireland, identify pathways through rehabilitation for survivors, and document the experience of family caregivers. The ms is clearly written and extremely interesting, and the study protocol seems compelling and likely to result in valuable contributions.

  A limitation of the ms is that it is only a description of the study protocol, with no findings. It strikes me as a very strong grant proposal, more than a journal publication. The journal editors will have to determine whether it fits within their journal’s purview to present a study protocol only.

 The most likely limitation of the eventual study seems to be that its findings will be limited to Ireland and perhaps not relevant to other countries. Even so, the fact that is will be a nation-wide study helps to argue in favor of the generalization of its findings.

 The fact that it will include a focus on family caregivers, using a good measure of burden, will be an important strength of the study.

 The exclusion of individuals with mild TBI (mTBI) strikes me as unfortunate. Worldwide, the large majority of persons with TBI have a mTBI, as the authors point out, and many of them will continue to experience TBI-related symptoms for years after the injury. It seems like a missed opportunity to follow many individuals some of whom will not completely recover.

 Some points of clarification are needed. Some individuals with TBI may not have a family caregiver or whose caregiver will not be willing to participate in the study. It was not clear whether individuals without a willing family member will still be eligible for the study. If they are excluded, this may very well bias the sample. If they are included, the sample size for caregivers will be smaller and statistical power weaker.

 Outcome measures for persons with TBI include three measures of quality of life. It wasn’t clear why three such measures (which cover much of the same territory) were selected or how distinct findings will be interpreted.

Is the study design appropriate for the research question?

Yes

Is the rationale for, and objectives of, the study clearly described?

Yes

Are sufficient details of the methods provided to allow replication by others?

Yes

Are the datasets clearly presented in a useable and accessible format?

Not applicable

Reviewer Expertise:

TBI, family caregiving, rehabilitaiton

I confirm that I have read this submission and believe that I have an appropriate level of expertise to confirm that it is of an acceptable scientific standard, however I have significant reservations, as outlined above.

# Response

**Published:** 2021-08-13

**Authors:** Anthony Staines

**Affiliations:** Dublin City University, Ireland

We thank the reviewer for her helpful comments.

 This paper is precisely a study protocol, as the title announces, and hence has no results.

 It is indeed unfortunate that we are not able to include mild head injury in our work, but our resources are very limited, and moderate to severe head injury is the main driver of needs for rehabilitation, and the main focus of our charity co-funders.

 The comment about outcome measures is well taken, and indeed our final choice of measures was the product of much discussion - we are partly motivated by what is achievable, and partly by a desire for consistency with the existing literature. We accept that there were many other possible choices, and were we doing the study again, we might make other choices.
